# Chiral Bicomponent Melamine/Amino Acid Eutectogels: Efficient Catalysts for the Aldol Reaction

**DOI:** 10.1002/cssc.70751

**Published:** 2026-05-22

**Authors:** Salvatore Marullo, Paolo Reale, Francesca D’Anna

**Affiliations:** ^1^ Dipartimento STEBICEF Università degli Studi di Palermo Palermo Italy

**Keywords:** aldol reaction, bicomponent supramolecular gels, catalysis, eutectogels

## Abstract

Supramolecular gels, characterized by reversibility, stimuli responsiveness, and high surface area, represent a promising class of materials in catalysis. In this work, we explored the possibility to obtain supramolecular melamine/amino acid‐based eutectogels, and we tested them as heterogeneous catalysts for the aldol reaction between *p*‐nitrobenzaldehyde and cyclohexanone. Gels were characterized by determining the critical gelation concentration and gel‐sol transition temperature. Mechanical features of soft materials were assessed performing strain and frequency sweep measurements, and their self‐sustaining properties were also investigated. Morphology of gel phases was analyzed by scanning electron microscopy. Resonance light scattering investigation was carried out to study gelation process and to investigate the size of aggregates featuring gel phases. Fluorescent behavior in gel phase and in solution was also studied, suggesting that gel formation was driven by an aggregation enhanced emission (AEE) process. Performing the aldol reaction, the experimental conditions were optimized in terms of reaction time, temperature and amount of catalyst. Under the best experimental conditions, we obtained 73% of yield and 76–24 anti/syn *diasteroisomeric* ratio with 75% of *enantiomeric excess*_anti_. Products were easily separated with simple extractions with a green solvent (ethyl acetate), and gel phase was recycled at least five times without loss in stereoselectivity.

## Graphical abstract

Chiral bicomponent eutectogels were used as confined reaction media for stereoselective aldol reaction between cyclohexanone and *p*‐nitrobenzaldehyde, proving efficient and reusable catalysts.

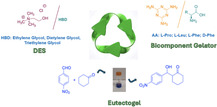

## 1 Introduction

Chirality plays a crucial role in nature at different levels. It describes the property of an object that cannot be superimposed on its own mirror image, a feature exhibited by many molecules. At the biological level, nature prefers a particular type of chirality selecting, for example, L‐amino acids as building blocks of the proteins, or D‐sugars as component of DNA or RNA. By a pharmaceutical point of view, many molecules are chiral; but often, only one enantiomer is pharmacologically active or safe; the other one may be inactive, less effective, or even toxic [1, 2].

For the above reasons, controlling stereoselectivity during a chemical reaction is extremely important. Stereoselectivity is defined as the preferential formation of one stereoisomer over another in a chemical reaction [3]. In particular, carbon–carbon (C–C) bond formation reactions (such as Michael addition, Friedel–Crafts alkylation, aldol, Mannich, and Diels–Alder reaction) often lead to asymmetric molecules that are essential to the synthesis of many molecules of pharmaceutical interest such as monoterpenes, indoles, benzylisoquinoline alkaloids [4], and so on.

One of the principal strategies to control stereoselectivity in chemical reactions is the use of a chiral catalyst.

In this context, enzymes represent a valuable class of chiral catalysts able to act in mild reaction conditions, with substrate specificity and biodegradability. Consequently, enantioselective formation of C–C bonds catalyzed by hydroxynitrile lyases (HNLs), benzaldehyde lyases (BALs), transketolases, aldolases, or decarboxylases have been used as efficient and versatile tools for asymmetric syntheses [5].

During the years, the good performance of enzymes in stereoselective catalysis has served as a natural model for the design of supramolecular catalysts. Indeed, one of the goals of supramolecular chemists is to create systems in which a receptor or a cavitand is connected to an active site, mimicking enzyme catalysis and generally involving typical reactions that are carried out by enzymes [6].

This explains the surge of interest that has been recently addressed to the development of nanoreactors, in which different functional groups are incorporated into coordination cages during the self‐assembly process, opening new frontiers. Confined cavities, in analogy to an enzyme's binding pocket, enable substrate recognition, transition‐state stabilization, and controlling of product release. The combination of chirality and confined spaces in such supramolecular coordination cages creates conditions to promote enantioselective transformation useful in asymmetric catalysis [7].

An interesting class of “supramolecular cages” can be represented by supramolecular gels, typically composed of low molecular weight gelators (LMWGs) self‐assembled through noncovalent interactions in a solvent, creating a 3D‐fibrous network [8, 9]. The solid‐like appearance and mechanical properties of these materials result from the immobilization of the liquid (major component) into the interstices of a self‐assembled solid matrix (minor component), due to surface tension and capillary forces [10]. Thus, supramolecular gels for their reversible nature, are characterized by high specific surface areas, good diffusion properties, and responsiveness to external stimuli [11].

The above properties endow them of interesting features, which are absent in conventional heterogeneous catalytic systems. Consequently, self‐assembled LMWGs can act as dynamic, nanostructured media for controlling chemical reactions, offering benefits like enhanced catalysis, tailored selectivity, and protection of sensitive species, essentially creating “nanoreactors” with tunable properties [12].

In general, supramolecular gels based on LMWGs and used as catalysts are mainly represented by hydrogels [13, 14]. Conversely, few examples have been reported in literature of catalytic gels systems based on nonconventional solvents such as ionic liquid and deep eutectic solvent (DES) [12, 15, 16]. In particular, the ones based on DES, the so called eutectogels, offer a lot of advantages, as solvents used are easy to prepare and are characterized by low cost, low volatility, high biodegradability, and, in general, absence of toxicity [17].

Among stereoselective reactions, aldol reaction holds a central role in organic chemistry because it is a fundamental carbon–carbon bond‐forming reaction with broad applications in the synthesis of complex molecules, including pharmaceuticals products. Among catalysts able to promote this reaction, L‐proline (L‐Pro) is well known for its ability to act as a bifunctional organocatalyst. Indeed, its amino functional group acts as a Lewis base, while the carboxylic acid acts as Brønsted acid catalyst [18]. Consequently, L‐Pro‐based supramolecular gels have recently offered a good way to perform aldol reaction with high yield and stereoselective discrimination [14, 16].

**SCHEME 1:**
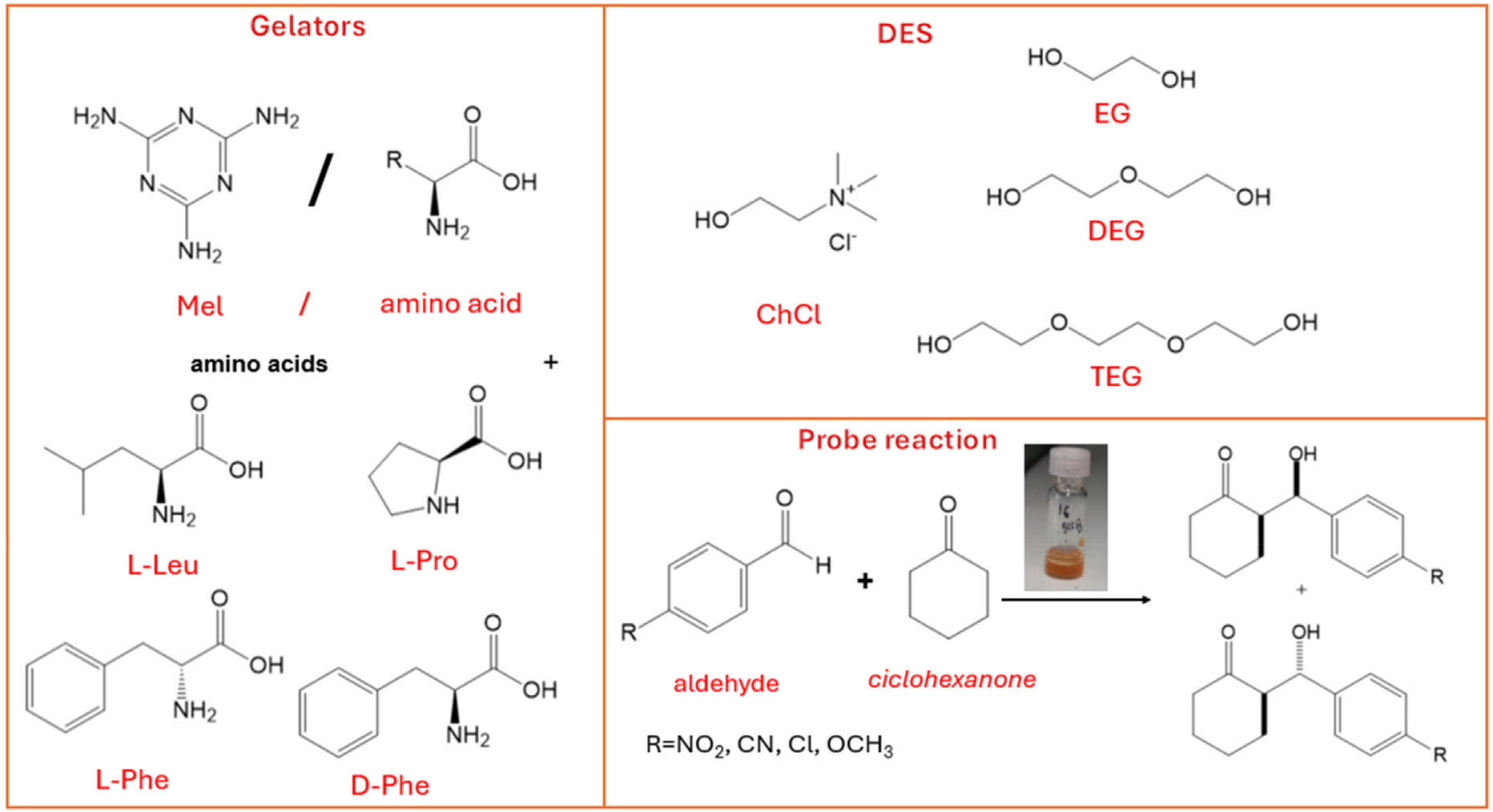
Structure of gelators and deep eutectic solvents used; schematic representation of aldol reaction.

In the light of the above premises, in this work, we explored the possibility to obtain bicomponent catalytic eutectogels, conjugating the aspect of sustainability given by DES and the presence of amino acids, the most used and simple catalysts for this reaction, as cogelators (Scheme 1).

Gelators consisted of composites of melamine (Mel) and amino acids. Mel is widely used in combination with formaldehyde to produce thermosetting resins, applied in paints, wood binders, and food delivery industries [19, 20]. Their wide application has significantly boosted the problem of plastic pollution in the above sectors, and recently, several efforts have been devoted to the possibility to perform their degradation, obtaining Mel as intermediate [21]. From a structural point of view, Mel shows a very high symmetry, and the presence of both hydrogen bond donor and acceptor sites could favor the formation of intermolecular interactions needed for the growth of the gel network [22, 23]. On the other hand, Mel‐based systems have been already used as catalysts for Knoevenagel condensation and in combination with adipic acid for cascade deacetalisation– Knoevenagel condensation [4, 24]. In our case, its combination with a chiral comonomer, like an amino acid, could give rise to the obtainment of a chiral gelator exhibiting catalytic activity. On the other hand, advantages in using amino acids as cogelators also derive from their sustainable origin, as they can be obtained from protein waste [24, 25].

Gelation tests were carried out in different DES with choline chloride as hydrogen bond acceptor and glycols as hydrogen bond donor. We used cholinium chloride/triethylene glycol (1:3) (ChCl/TEG), cholinium chloride/diethylene glycol (1:3) (ChCl/DEG) and cholinium chloride/ethylene glycol (1:2) (ChCl/EG) (Scheme 1). The amino acids investigated were L‐Pro, L‐leucine (L‐Leu), L‐phenylalanine (L‐Phe), and D‐phenylalanine (D‐Phe). They were chosen for the different side‐chain structure that could affect gel properties and catalytic activity.

For each gel, we determined the critical gelation concentration (CGC) and gel‐sol transition temperature (*T*_gel_). Gelation time and the size of aggregates were studied performing resonance light scattering (RLS) investigations. In addition, rheological investigation and self‐sustaining tests were carried out. Fluorescence properties in gel phase and in solution were also analyzed.

Eutectogels were used as heterogeneous catalysts and reaction media for aldol reaction between *p*‐nitrobenzaldehyde and cyclohexanone (Scheme 1). The reaction was carried out at 25°C and at 4°C, changing the reaction time. The best performances in terms of conversion and yield were obtained with L‐Pro‐based gel. This is the reason why this system was used to evaluate the possibility of reusing and to investigate both substrate scope and loading. Data collected evidence that Mel/L‐Pro eutectogel allowed to perform the aldol reaction with high conversion and yield and good diastereo‐ and enantioselectivity ratios. This system was reusable for, at least, five times without significant loss in performance. After the third cycle, notwithstanding the loss in yield, stereoselectivity stayed constant. Furthermore, it can be used with a very high substrate loading without loss in performance.

## 2 Results and Discussion

### 2.1 Gelation Tests and Eutectogel Characterization

To carry out gelation tests, composites of Mel and amino acids were prepared by grinding in a mortar the suitable amounts of the components to obtain a homogeneous powder. Gels were generally prepared by weighing into a screw‐capped vial (diameter 1 cm) the suitable amounts of composite and 250 mg of DES. The sample vial was heated at 100°C until a clear solution was obtained. Then, it was stored at room temperature for one night. Gel phase formation was verified by the tube inversion test [26]. Various gelation tests were performed by decreasing the concentration of gelator, with the aim to determine the CGC (i.e., the lowest amount of gelator able to form gel phase). For a useful comparison, gelation tests were also performed using Mel as gelator. On the other hand, gel‐sol transition temperature (*T*_gel_) were determined through the falling drop method [27].

Results of gelation tests are reported in Table S1, whereas CGC and *T*_gel_ for gels obtained in ChCl/TEG are reported in Table 1.

**TABLE 1:** CGC and *T*_gel_, at 5% wt, for Mel/amino acid in eutectogels ChCl/TEG.

Gelator	CGC, % wt	*T*_gel_ at 5% wt, °C^a^
**Mel**	**4**	**34**
**Mel/L‐Leu 2:1**	**3**	**51**
Mel/L‐Leu 1:1	4	47
Mel/L‐Leu 1:2	5	39
Mel/L‐Leu 1:3	8	34^b^
**Mel/L‐Pro 2:1** Mel/L‐Pro 1:1 **Mel/L‐Phe 2:1** Mel/L‐Phe 1:1 **Mel/D‐Phe 2:1**	**3** 4 **3** 4 **3**	**52** 35 **42** 38 **44**

a *T*_gel_ were reproducible within ± 1°C.

b *T*_gel_ was determined at 8% wt.

Analysis of data reported in Table 1 and S1 evidences that Mel formed white and opaque eutectogels in ChCl/DEG and ChCl/TEG, with CGC increasing on going from ChCl/DEG to ChCl/TEG (Table S1). Adding amino acid allowed the obtainment of a series of orange‐colored eutectogels at different molar ratios. In particular, in ChCl/EG, only Mel/L‐Phe was able to give gel phase formation with CGC values decreasing by increasing the amino acid amount (see Table S1, 8 and 5 wt% for Mel/L‐Phe 1:1 and Mel/L‐Phe 1:3, respectively). Differently, analysis of CGC values collected for gels obtained in ChCl/TEG shows that the above parameter increased in parallel with the amount of amino acid. Indeed, composite Mel/L‐Leu 1:1 form a gel at 4 wt% wt, whereas the one at 1:3 ratio required at least 8% wt of gelator to form a gel phase. In this solvent, L‐Pro and L‐Phe showed an analogous trend, as CGC increased from 3 to 4 wt% moving from composite at 2:1 to 1:1 ratio, and the further increase in the amino acid amount disfavored gelation process.

Mel/L‐Leu was the only composite able to form soft materials also in the presence of excess in amino acid, as accounted for by gel phase formation at 1:2 and 1:3 Mel/amino acid ratio. However, the increase in amino acid amount induced a parallel increase in CGC values (see Table 1, 4 and 8 wt% for Mel/Leu 1:1 and 1:3, respectively).

Comparison between CGC values obtained for Mel/L‐Phe in ChCl/EG and ChCl/TEG allows evidencing the role played by the nature of the HBD component of the DES, as testified by the significant decrease in CGC detected going from the former to the latter DES (8 and 4% wt in ChCl/EG and ChCl/TEG; *cfr* Tables 1 and S1).

To better rationalize why only in the presence of L‐Phe a gel is obtained in ChCl/EG, while no gel formation occurs in ChCl/DEG, we analyzed the gelling ability of the single amino acids, in the DES considered. Gelation tests revealed that no gel was formed by L‐Leu in all the DES considered. Conversely, in the presence of L‐Phe, a gel was obtained in ChCl/EG, at 6 wt%. The higher gelling ability of L‐Phe compared with the other amino acid is consistent with literature reports [28]. In addition, the superior gelling ability of L‐Phe is also consistent with the reduction in CGC on increasing the amount of amino acid in the composite. In ChCl/DEG, no gel formation was observed even in the presence of L‐Phe, but only clear solutions. Consequently, we attribute the absence of gelation detected in ChCl/DEG to the solubility of the components, which is too high to support gelation.

Trend of *T*_gel_ values perfectly agrees with the one of CGCs, as the thermal stability decreased as the amount of amino acid in the composite increased. Indeed, at 2:1 ratio, gels were more thermally stable and showed a lower CGC. In the above conditions, CGC values were not affected by the different side chain structure, being equal to 3% wt in all cases. Differently, *T*_gel_ values gradually decreased going from aliphatic to aromatic side chain (*T*_gel_ = 51°C, 52°C, 42°C, and 44°C for L‐Leu, L‐Pro, L‐Phe, and D‐Phe respectively, Table 1).

In all cases, bicomponent eutectogels proved to be more stable than Mel‐based gel phases. Furthermore, all gels were thermoreversible, and they resulted stable for at least 6 months at room temperature, as assessed by tube inversion test. Considering the higher stability of eutectogels formed by Mel/amino acid at 2:1 ratio, they were used for characterization and from now they will be indicated simply as Mel/amino acid.

Eutectogels at 5% wt were characterized from a mechanical point of view, performing frequency and strain sweep investigations, to analyze their viscoelastic nature.

In strain sweep measurement, the variation of elastic (*G*′) and viscous modulus (*G*″), as function of the percentage of strain, showed an intermediate behavior between solid and liquid materials, typical of the gels. Indeed, at low percentages of strain *G*′ was higher than *G*″, showing a solid‐like behavior, but the increase in the strain percentage gave rise to the modulus inversion with *G*″ values higher than *G*′, characteristic of the liquid‐like behavior. This trend allowed to detect the crossover point of the moduli (*γ*) that account for the strain that should be applied to induce the gel network breakdown.

The gel‐like nature of eutectogels was further confirmed by frequency sweep measurements, in which, at a fixed strain (0.02%), *G*′ was higher than *G*″, with both moduli fairly independent from the angular frequency.

**FIGURE 1:**
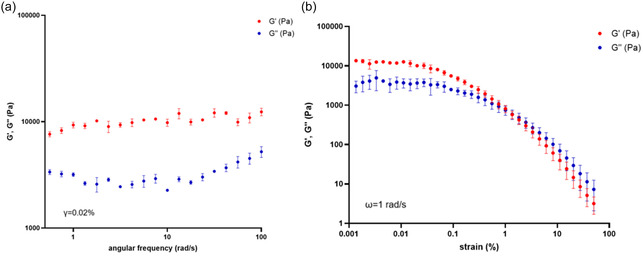
Rheological investigation for Mel/L‐Leu eutectogel in ChCl/TEG at 5 wt%. (a) Frequency sweep at *γ* = 0.02% in the range 0.1–100 rad/s. (b) Strain sweep at *ω *= 1 rad/s in the range 0.001%–100%.

In Figure 1, plots of strain and frequency sweep measurements for Mel/L‐Leu eutectogel are reported, whereas data corresponding to all the other gel phases are displayed in Figure S1.

In Table 2, rheological parameters for all gels investigated are reported.

**TABLE 2:** Values of *G*′, *G*″, and *γ* (cross point) for eutectogels at 5 wt%. Values at *γ* = 0.02% and *ω* = 1 rad/s. Error limits are based on the average of three different measurements with different aliquots.

Gel	***G*** ′, **Pa**	***G*** ″, **Pa**	tan*δ*	***γ***_**c,**_ **%**
Mel/L‐Pro	22,000 ± 1000	9600 ± 800	0.43 ± 0.01	0.48 ± 0.07
Mel/L‐Leu	9600 ± 600	3100 ± 200	0.32 ± 0.04	1.6 ± 0.2
Mel/L‐Phe Mel/D‐Phe	24,000 ± 2000 22,000 ± 1000	11 000 ± 500 15 000 ± 1000	0.46 ± 0.06 0.63 ± 0.08	0.88 ± 0.13 0.6 ± 0.2

*G′* values account for the stiffness of the gel phase, and, in our case, with the only exception of Mel/L‐Leu, all eutectogels investigated show comparable values. Besides *G′* and *G″*, Table 2 also shows tan*δ* and *γ*_c_ values. tan*δ* is a measure of the strength of colloidal forces operating inside the gel network. In general, values much lower than 1 are indicative of the occurrence of very intense colloidal forces. Analysis of data collected in the table shows that Mel/L‐Leu eutectogel exhibited the lowest value of tan*δ* and was consequently characterized by the most intense colloidal forces. Accordingly, this gel also showed the highest value of *γ*_c_, indicating that the application of a higher strain was needed to induce the gel network breakdown.

Gel properties were also investigated by using RLS measurements. This technique is used to have information about aggregation of molecules containing chromophores. RLS measurements for eutectogels at 5 wt% were performed as a function of time. This kind of investigation allows to have information about gelation mechanism and time needed to have gel phase. Furthermore, the intensity of scattered light (*I*_RLS_) can be directly related to aggregate dimensions [15, 29].

**FIGURE 2:**
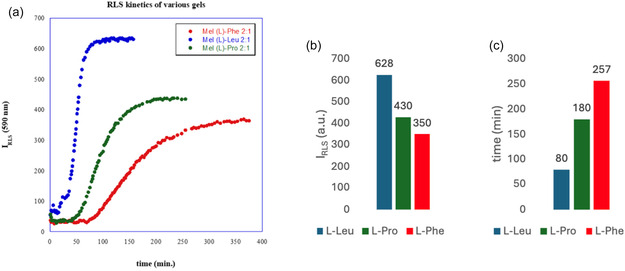
(a) Plot of *I*_RLS_ as a function of the time for Mel/amino acid eutectogels in ChCl/TEG at 5 wt%. (b) *I*_RLS_ value corresponding to gel phase formation. (c) Gelation time for different eutectogels.

Trends of *I*_RLS_ as a function of time are reported in Figure 2a, whereas in Figure 2b,c, *I*_RLS_ values at *λ *= 590 nm and gelation times are reported, respectively.

Analysis of RLS trends showed that gels formed in one step as *I*_RLS_ increased until reaching a constant value. This plateau region of the graph corresponds to gel formation, as once formed, aggregates maintained their dimensions without any further rearrangements. Analysis of kinetic trends reported in Figure 2 gives the following trend for gelation time: L‐Leu < L‐Pro < L‐Phe (Figure 2c) and the one: L‐Leu > L‐Pro > L‐Phe for the size of the aggregates featuring gel phases (Figure 2b). In general, gels with aliphatic amino acids (L‐Leu and L‐Pro) formed faster, and they showed larger aggregates. Considering the above parameter, the most significant changes were detected going from L‐Leu to L‐Pro, and this trend perfectly recalls the one of *γ*_c_ value. Indeed, Mel/L‐Leu eutectogel, featured by the presence of the largest aggregates, is also the soft material exhibiting the highest resistance to strain application.

**FIGURE 3:**
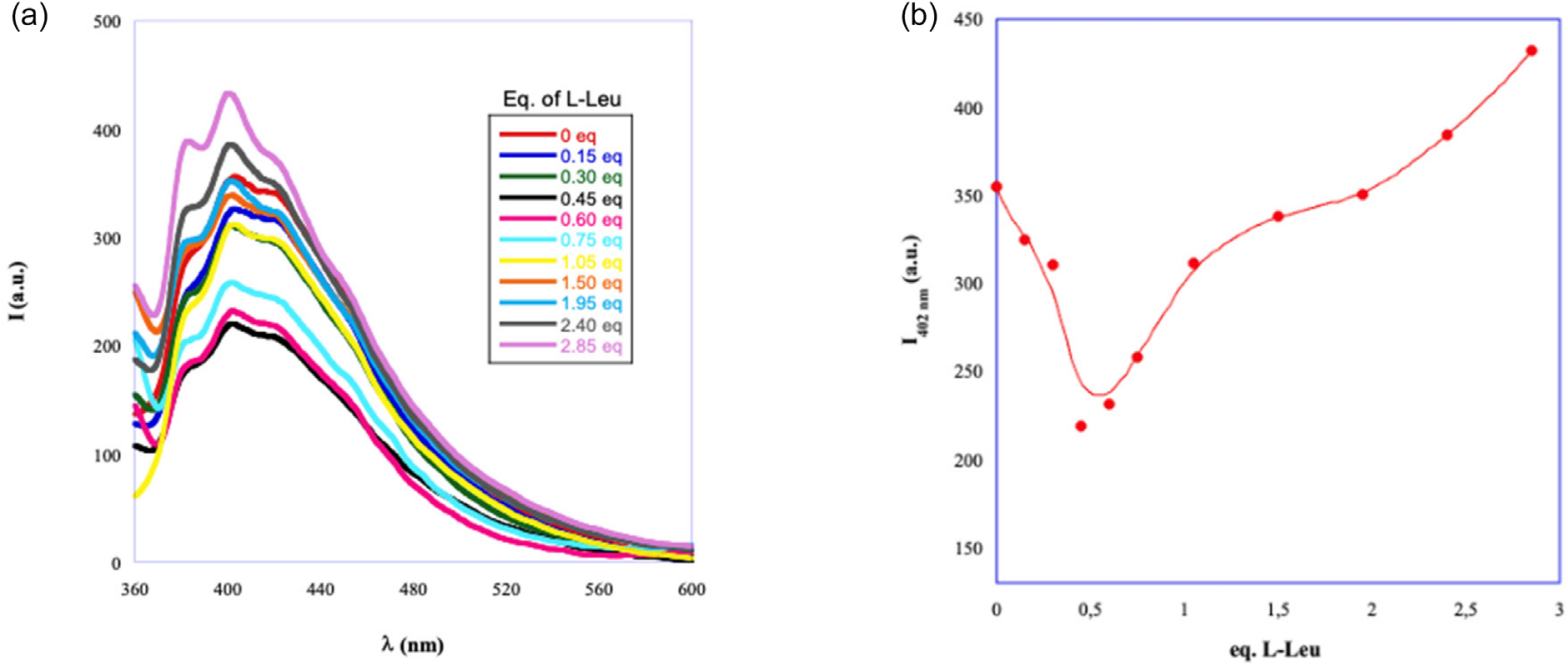
(a) Emission spectra of Mel 1 × 10^−5^ mol/g in ChCl/TEG as a function of equivalents of L‐Leu. (b) Trend of emission intensity at 402 nm (*I*_402_) as a function of equivalents of L‐Leu.

With the aim of determining the stoichiometry of the Mel/amino acid complexes acting as gelator, the effect of the amino acid concentration on Mel emission properties was studied. Mel shows interesting fluorescence properties in water and in organic solvents [30], but its emission behavior in DES has been rarely studied. To this purpose, different solutions having a fixed amount of Mel (1 × 10^−5^ mmol/g) and increasing concentrations of amino acid were prepared in ChCl/TEG. Emission spectra collected for Mel/L‐Leu system are reported in Figure 3, whereas spectra recorded in presence of the others amino acids are reported in Figures S2 and S3.

Data reported in Figure 3a evidence that emission intensity, as a function of L‐Leu equivalents, gave a nonmonotonic trend, with a change in slope at ~0.5 equivalents. This nonmonotonic trend induced us to suppose that amino acid actively participates in the building of the three‐dimensional network of the eutectogel and this could be classified as a case of bicomponent cogelation [31]. According to previous reports in literature [32], this indicated that in ChCl/TEG solution, Mel and L‐Leu interact to give a 2:1 complex. This result perfectly agrees with data collected determining gel‐sol transition temperature of gel phases that account for the highest thermal stability of the gel formed by the Mel/L‐Leu (2:1) composite (see Table 1). The further addition of amino acid increased Mel solubility in DES and decreased the thermal stability of the gel. Applying the same procedure to all composites allowed determining stoichiometric ratios reported in Table 3 (see also Figure S2).

**TABLE 3:** Stoichiometric ratios for Mel/amino acid complex determined by fluorescence investigation.

**Amino acid**	**Stoichiometry** **Mel/amino acid**
L‐Pro	2:1
L‐Leu	2:1
L‐Phe	1:1
D‐Phe	1:1

The formation of a complex is also consistent with the appearance of orange color during gel preparation. To lend further support to this hypothesis, we subjected the single component of the eutectogels, Mel and the amino acids, to the same treatment involved in gel preparation. In all cases, we did not observe any appearance of color, thus ruling out that the orange color may be due to early‐stage degradation.

Analysis of data reported in Table 3 evidences that the amount of Mel in the complex gradually decreases on going from aliphatic (L‐Pro and L‐Leu) to aromatic side chain (L‐ or D‐Phe). The above trend allows highlighting the relevance of interactions operating between Mel and amino acids in determining complex stoichiometry. Indeed, in all cases, gelator components can interact through hydrogen bond. However, only in the case of L‐ or D‐Phe, the complex is further stabilized by π−π interactions between the electron poor aromatic ring of Mel and the phenyl ring of the amino acid. This further interaction plays a pivotal role and induces a change in the stoichiometric ratio, which can also be related to the higher gelling ability of this amino acid, as mentioned earlier in this section.

**FIGURE 4:**
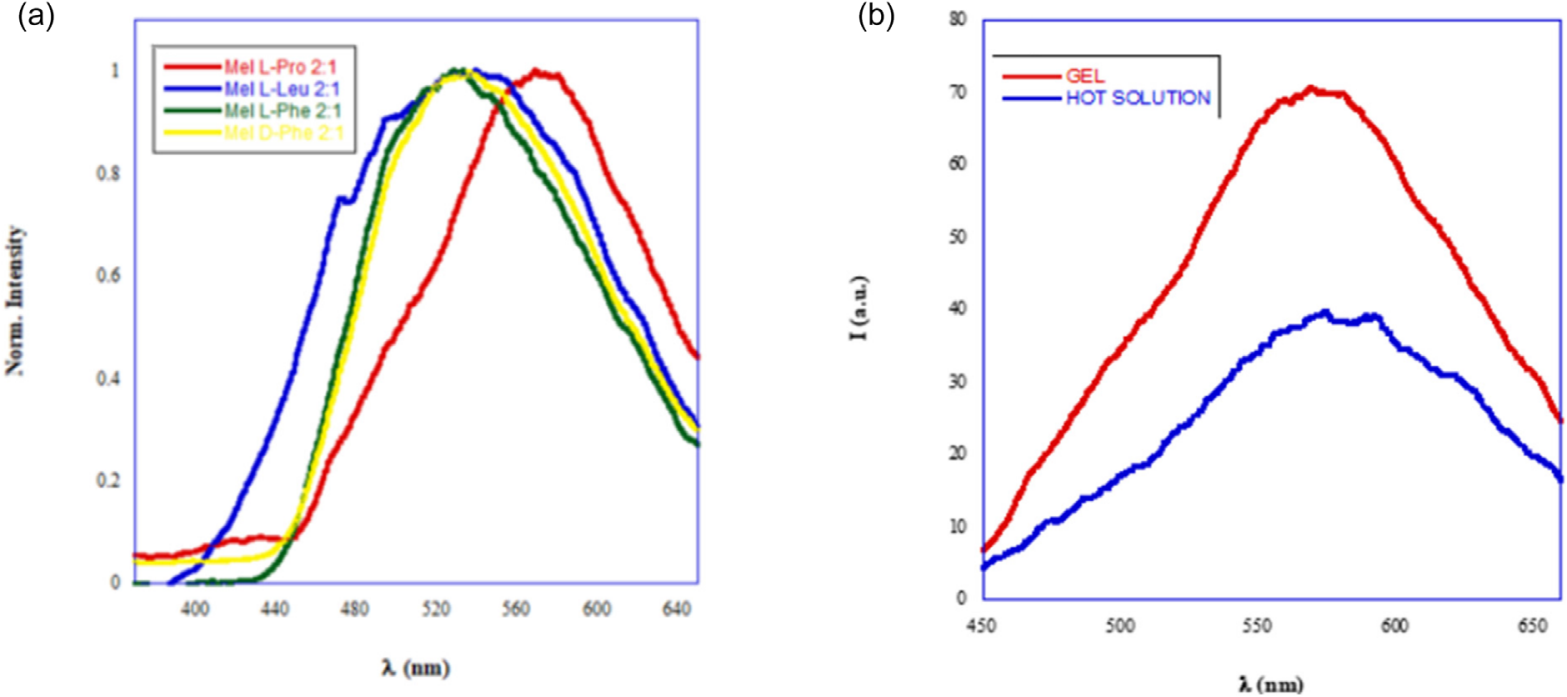
(a) Emission spectra of eutectogels at 5 wt% in ChCl/TEG. (b) Comparison between spectra of Mel/L‐Pro as eutectogel and, after heating, of the corresponding hot solution.

With the above results in mind, and to gain further insights on gel properties, their emission behavior as a function of the different nature of the composite was investigated. To this aim, we firstly recorded emission spectra of Mel eutectogel at 5 wt% (Figure S4) observing a band centered at 467 nm. Subsequently, emission spectra of different Mel/amino acid eutectogels at 5 wt% were acquired, and, after heating, the emission spectra of the corresponding hot solution were also recorded. In Figure 4a, normalized emission spectra of all eutectogels are reported, whereas in Figure 4b, the comparison between the emission behavior of Mel/L‐Pro eutectogel and of the corresponding hot solutions is displayed. Emission spectra corresponding to all the other eutectogels are reported and displayed in Figure S5.

The presence of the amino acid in the composite significantly changed the emission behavior of the eutectogels. Indeed, independent from the nature of the amino acid, bicomponent eutectogels exhibited a completely different shape of the band, which in all cases was batochromically shifted.

Indeed, the main emission band moved from 466 nm in Mel eutectogel to 572 nm in Mel/L‐Pro eutectogel (*λ*_max_: 572, 539, 534 nm D‐Phe, 531 nm for Mel/L‐Pro, Mel/L‐Leu, Mel/D‐Phe and Mel/L‐Phe, respectively). The magnitude of the batochromic shift gradually increased on going from aromatic to aliphatic amino acid. The above result supports the hypothesis that bicomponent eutectogels are the result of a cogelation process, in which the “true” gelator is the complex formed in the presence of the amino acid.

Interestingly, the comparison between the emission behavior of eutectogels and corresponding hot solutions evidences that, in all cases, gelation occurs through aggregation‐enhanced emission (AEE) process, as accounted for by the increase in emission intensity on going from the hot solution to the corresponding gel phase. We previously detected a similar behavior, studying properties of some 1,8‐naphthalimide‐based hydrogels, used as theranostic agents [33, 34]. In general, this kind of behavior proves to be quite interesting as such materials can be applied in different fields spanning from engineering to biomedicine [35].

**FIGURE 5:**
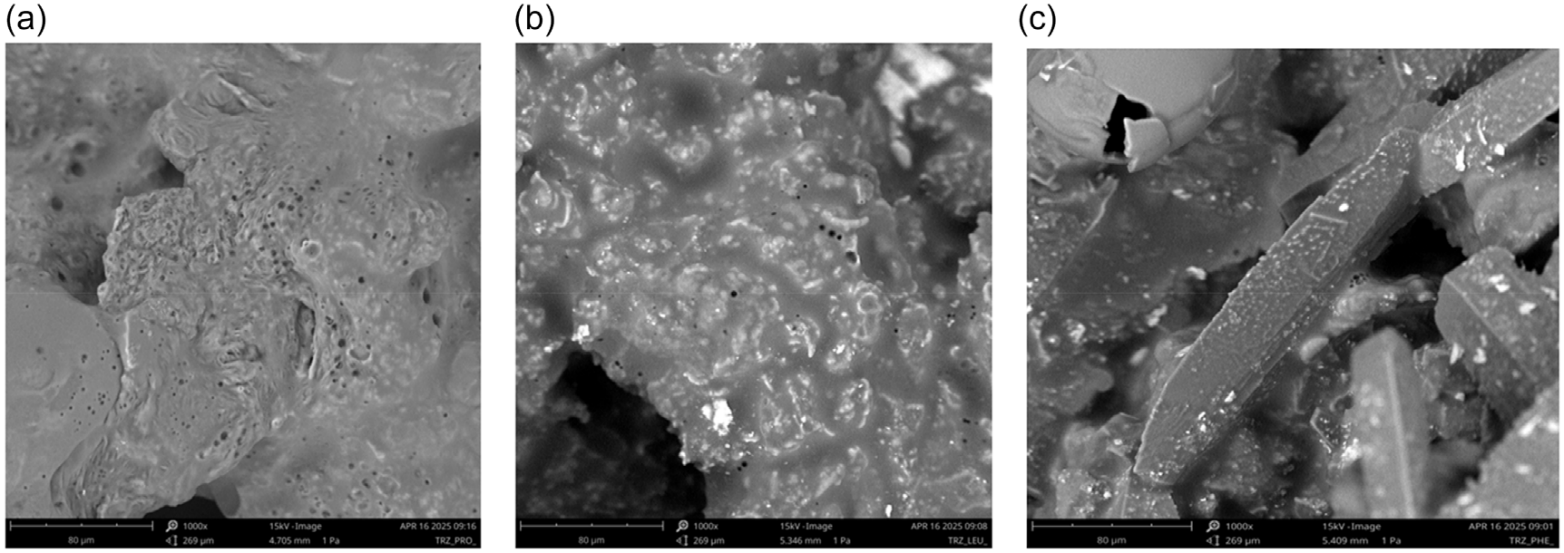
SEM images of xerogel at 5 wt%. (a) Mel/L‐Pro 2:1. (b) Mel/L‐Leu 2:1. (c) Mel/L‐Phe 2:1.

Eutectogels were also analyzed from a morphological point of view, performing SEM investigation on the xerogels obtained after washing gel phases with a small amount of ethyl acetate and drying at room temperature. We know that the organization in gel and xeroegel phases can be different because of the solvent removal. However, as all samples were subjected to the same experimental procedure, we are confident that, phase being constant, the comparison among materials can be considered homogeneous. SEM micrographs, at 1000x magnification, are displayed in Figure 5.

Analysis of SEM images evidences the significant effect that the different nature of amino acid exerts on gel morphology. Indeed, compact three‐dimensional networks were collected in the presence of aliphatic amino acids (Figure 5a,b). However, compactness degree decreased going from L‐Pro to L‐Leu, whereas Mel/L‐Phe eutectogel shows a completely different morphology characterized by the presence of cylindrical shaped aggregates. On this light, the above result can be related to the size and shape of the amino acid that determines the stoichiometry of Mel/amino acid complex in the gelator (see above) and, consequently, also the network organization. Changes in morphology prove to be related to the amount of amino acid present in the fluorescent complex of the gelator but also the nature of the side chain that determines the nature of the interactions. Results collected seem to indicate that, if hydrogen bond is the prevalent interaction, a regular and compact network was formed. Once π–π interactions become operative, the stacking possibility among cogelator complexes gives rise to the formation of more discrete objects.

### 2.2 Catalytic Activity of Eutectogels

After gel phase characterization, these materials were investigated for their potential catalytic activity. To this aim, the aldol reaction between cyclohexanone and *p*‐nitrobenzaldehyde was used as probe reaction. In details, 250 mg of gel was prepared at 5 wt% in a 1 cm diameter vial. In the first attempt, the reaction was performed at 25°C in an orbital shaker, at 130 rpm and employing a ketone/aldehyde molar ratio (*n*_K_/*n*_A_) equal to 10.

The reagents were added on the top of a preformed gel, and the reaction was carried out for 20 h. At the end of the reaction, the gel nature of the samples was verified, using the tube inversion test. In Table 4, conversions, yields, and diastereomeric (*dr*) and enantiomeric ratios (*ee*) are reported.

**TABLE 4:** Conversions, yields, and diastereomeric and enantiomeric excess ratios for the aldol reaction between cyclohexanone and *p*‐nitrobenzaldehyde (*n*_K_/*n*_A_: 10) performed in gel phase at 5 wt%, at 25°C, for 20 h in orbital shaker.

Entry	GEL (5% wt)	Conversion, %^a^	Yield, %^a^	Diastereomeric ratio^b^ (ANTI/SYN)	Enantiomeric excess^b^ (ANTI/SYN)
1	Mel/L‐Pro 2:1	85	73	59/40	47/12
2	Mel/L‐Leu 2:1	38	26	59/41	78/58
3	Mel/L‐Phe 2:1	27	20	51/48	35/13
4	Mel/D‐Phe 2:1	34	30	57/43	68/53
5	L‐Pro in solution	48	42	73/27	90/17

a Yield and conversion of the reaction are obtained after purification by chromatographic column; values were reproducible within ± 3%.

b Diastereomeric ratio and enantiomeric excess were determined by using an HPLC with a chiral stationary phase.

It is noteworthy that, for a useful comparison, the reaction was also carried out using Mel eutectogel at 5 wt%, under the same experimental conditions, but without success. This indicated the pivotal role played by the amino acid in the catalytic process.

Analysis of data collected evidences that, in all cases, the eutectogel environment favored the outcome of the reaction with good selectivity. Catalytic activity was affected by the nature of the gelator, as both conversions and yields decreased along the order: Mel/L‐Pro> Mel/L‐Leu> Mel/D‐Phe> Mel/L‐Phe. Chirality of amino acid had a marginal effect on conversion and yield values, but a significant effect on the enantiomeric excess that increased from 35% up to 68% going from Mel/L‐Phe to Mel/D‐Phe. From a stereochemical point of view, in all cases, we detected the predominance of the *anti*‐stereoisomers, with the higher *ee* obtained in the case of Mel/L‐Leu (78%).

Since the best catalytic performance in terms of conversion and yield was obtained with L‐Pro‐based eutectogel, we evaluated the performance of this amino acid in solution of DES, collecting significantly lower conversion and yield values (*cfr* Entries 1 and 5 of Table 4). This testifies that the confined eutectogel microenvironment exerted a positive effect on the outcome of the reaction. A similar behavior has been previously detected studying the aldol reaction in L‐Pro‐based eutectogels, and collected results were ascribed to rigidification of aggregates in the gel network [16]. On the other hand, the catalytic positive effect, deriving from the structural organization of the gel network, was also detected studying the alcoholysis of anhydrides, catalyzed by cinchona alkaloid derivatives in ionic liquid gels [36].

**FIGURE 6:**
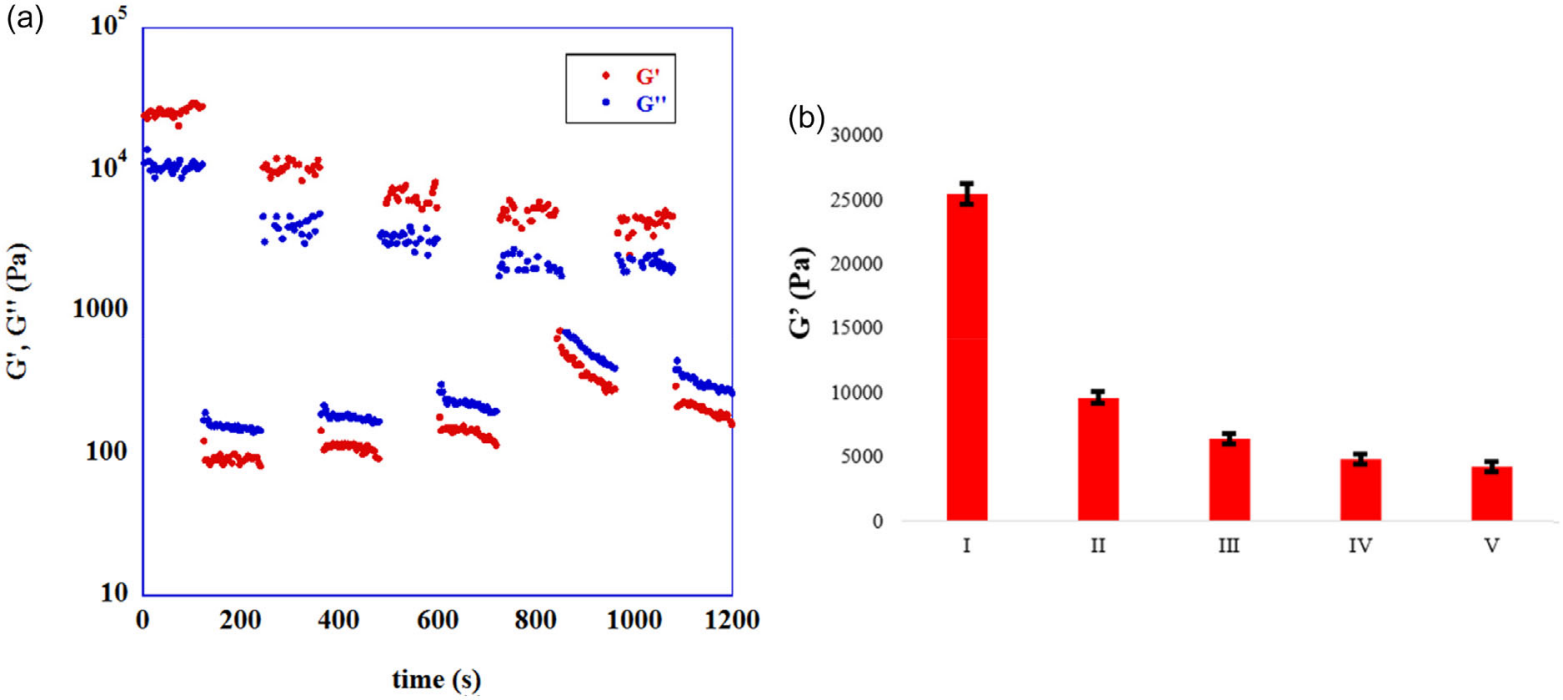
(a) Trends of *G*′ and *G*″ at 25°C as a function of time, after the application of nondisruptive (*γ* = 0.02%) and destructive strain (*γ* = 20%) to Mel/L‐Pro eutectogel at 5 wt%. (b) Plots of *G*′ values during under nondestructive regime at each cycle.

With the above results in mind, we took in consideration the possibility to optimize catalytic performance of the best system, namely, Mel/L‐Pro eutectogel. Under this light, we further investigated its properties analyzing its possible thixotropic and self‐sustaining behavior. With the aim to analyze the thixotropic behavior, eutectogel was subjected alternatively to disruptive and nondisruptive strain, to promote gel reformation after the application of a mechanical stimulus. In Figure 6a, trends of *G*′ and *G*″, after the application of the mechanical stimulus is reported, whereas in Table S2 the recovery percentages of *G*′, after each breakdown‐recovery cycle, are displayed. In Figure 6b, average values of *G*′, during under nondestructive regime at each cycle, are reported.

The gel nature of the sample was kept for five cycles, as accounted for by percentage of recovery in Table S2. After the first cycle, *G*′ decreased regularly up to the third one, after which its values stay practically constant.

**FIGURE 7:**
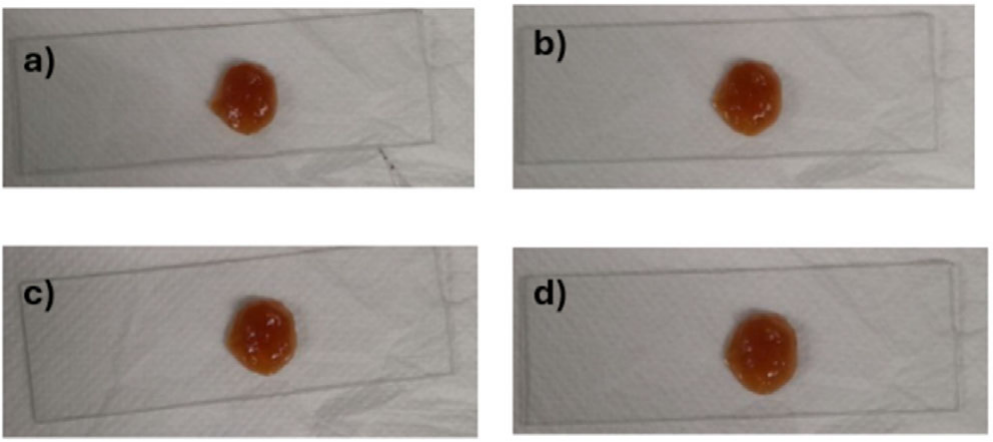
Self‐sustaining test for Mel/L‐Pro eutectogel at 5% wt in ChCl/TEG: (a) freshly deposited gel phase, (b) after 3 h, (c) after 6 h, and (d) after 9 h.

Mel/L‐Pro eutectogel was also investigated for its possible self‐sustaining nature. This property refers to the ability of gel phase to stand by itself without any support, retaining its shape. Self‐sustaining ability has been reported for metal organogels [37] and ionogels [38], but it has been poorly studied for eutectogels. To this aim, the eutectogel was prepared in a blister, and after 24 h, it was pulled out from the container and deposited on a glass plate. In the above conditions, it was able to retain its shape for at least 9 h (Figure 7).

After assessing the thixotropic nature of the eutectogel, we went on to improve the performance of the aldol reaction from a stereochemical point of view. To this aim, we analyzed the temperature effect, carrying out the reaction in a fridge, at 4°C. In this case, different from the reaction carried out in the orbital shaker, the process was performed without stirring. Data collected are reported in Table 5.

**TABLE 5:** Conversions, yields, and diastereomeric and enantiomeric excess ratios for the aldol reaction between cyclohexanone and *p*‐substituted benzaldehydes (*n*_K_/*n*_A_: 10) performed in gel phase at 5 wt%, at 4°C without stirring.

Entry	Phase	Substrate	Reaction time	Conversion, %^a^	Yield, %^a^	Diastereomeric ratio^b^ (ANTI/SYN)	Enantiomeric excess^b^ (ANTI/SYN)
**1**	Gel Mel/L‐Pro 2:1	*p*‐NO_2_	20 h	75	65	70/30	68/30
**2**	Gel Mel/L‐Pro 2:1	*p*‐NO_2_	24 h	84	73	74/26	75/25
**3**	Gel Mel/L‐Pro 2:1	*p*‐CN	24 h	52	49	91/9	72/12
**4**	L‐Pro in solution	*p*‐NO_2_	24 h	44	32	84/16	93/40

a Yield and conversion of the reaction are obtained after purification by chromatographic column; values were reproducible within ± 3%.

b Diastereomeric ratio and enantiomeric excess were determined by using an HPLC with a chiral stationary phase.

Using the same experimental conditions applied at 25°C, we detected better performance in terms of stereoselectivity (*ee*_anti/syn_: 47/12 and 68/30 at 25°C and at 4°C, respectively), but with a slight decrease in conversion and yield values. Increasing reaction time to 24 h allowed to obtain conversion and yield values comparable to ones got at 25°C, but with a significant increase in both diastereomeric and enantiomeric ratios (*cfr* Entry 1of Table 4 and Entry 2 of Table 5).

For a useful comparison, the reaction was also carried out in DES solution, using the same amount of L‐Pro contained in the gel. Once again, data collected testify the good performance of L‐Pro solution from a stereochemical point of view, but with much lower efficiency in terms of conversion and yield. The above results confirm the advantages deriving from proximity effects occurring in confined reaction media, such as gel phases.

Using the best experimental conditions, we explored the substrate scope, changing the nature of *p*‐substituted benzaldehydes, considering both electrons withdrawing (NO_2_, CN, and Cl) and electron donor (OCH_3_) substituents (Table 5). The reaction did not occur in the presence of the *p*‐clorobenzaldehyde and *p*‐methoxybenzaldehyde, featured by the presence of weak electron withdrawing and electron donor substituents, respectively.

As for the electron‐withdrawing substituents, analysis of data collected demonstrates that, in our experimental conditions, conversion and yield decreased from *p*‐nitrobenzaldehyde to *p*‐cyanobenzaldehyde, according to the decrease in the electronic effect.

We further analyzed the substrate scope, using *p*‐nitrobenzaldehyde, as model substrate, and changing the nature of the ketone. This aims to analyze also the effect deriving from the steric hindrance on the carbonyl group. Together with cyclohexanone, the reaction was also carried out in the presence acetophenone and acetone. Results collected are reported in Table 6.

**TABLE 6:** Conversions, yields, and diastereomeric and enantiomeric ratios, for the aldol reaction between different ketones and *p*‐nitrobenzaldehyde (*n*_K_/*n*_A_: 10), performed in Mel/L‐Pro eutectogel at 5% wt, at 4°C, for 24 h, on rest.

Ketone	Conversion, %^a^	Yield, %^a^	Enantiomeric excess^b^
Cyclohexanone	84	73	75 (ANTI)
Acetophenone	61	54	86
Acetone	86	63	8

a Yield and conversion of the reaction are obtained after purification by chromatographic column; values were reproducible within ± 3%.

b Diastereomeric ratio and enantiomeric excess were determined by using an HPLC with a chiral stationary phase.

Analysis of results collected shows that trend in conversion changes along the following series: acetone ~ cyclohexanone > acetophenone, according to the increase in the steric hindrance. Good enantioselectivity ratios were collected in the case of cyclohexanone and acetophenone, but the above parameter significantly dropped in the case of acetone. This result perfectly agrees with data previously reported in literature about the positive effect played by the increase in steric hindrance on the stereoselectivity of the target reaction, because of the formation of more rigid, chair‐like transition state [39].

Taking into consideration the environmental impact of the process, we also attempted the reuse of the gel phase and investigated the reagent loading aspect, with the aim to minimize the amount of materials used and therefor reduce the waste generated at the end of the reaction. Consequently, we performed the reaction with the substrates that gave the best results (cyclohexanone and *p*‐nitrobenzaldehye), and at the end of the reaction, the reaction mixture was extracted using ethyl acetate. Then, the residual organic solvent was removed by evaporation under vacuum, and fresh reagents were added for the reuse. Results collected are reported in Table 7.

**TABLE 7:** Conversions, yields, and diastereomeric and enantiomeric ratios, after the gel phase reuse, for the aldol reaction between cyclohexanone and *p*‐nitrobenzaldehyde (*n*_K_/*n*_A_: 10): performed in Mel/L‐Pro eutectogel at 5 wt% at 4°C, for 24 h without stirring.

Cycle	Conversion, %^a^	Yield, %^a^	Diastereomeric ratio^b^ (ANTI/SYN; %)	Enantiomeric excess^b^ (ANTI/SYN; %)
I	84	73	74/26	75/25
II	84	72	79/21	75/23
III	81	70	77/23	77/34
IV	64	56	78/22	77/28
V	64	55	68/32	81/47

a Yield and conversion of the reaction are obtained after purification by chromatographic column; values were reproducible within ± 3%.

b Diastereomeric ratio and enantiomeric excess were determined by using an HPLC with a chiral stationary phase.

Collected data evidence that the eutectogel can be reused at least three times without loss in performance in terms of conversion and yield values and no significant effect on diastereo‐ and enantioselectivity. In the fourth cycle, a significant drop in yield was detected, but diastereo‐ and enantiomeric ratio stayed constant until the fifth cycle.

With regard to the initial the reagents loading, starting from the *n*_a_/*n*_L‐Pro_ used to evaluate the performance of Mel/L‐Pro eutectogel (3.8), we changed the above parameter in the range 1–23. This allowed evaluating two important factor: (i) the resilience of the eutectogel to substrate loading and (ii) the effect deriving from the change in *n*_a_/*n*_L‐Pro_ ratio, as increasing the loading of the aldehyde and keeping constant the eutectogel composition gave rise to a gradual decrease in the percentage of catalyst used. Results collected are reported in Table 8.

**TABLE 8:** Conversions, yields, and diastereomeric and enantiomeric ratios for the aldol reaction between cyclohexanone and *p*‐nitrobenzaldehyde (*n*_K_/*n*_A_: 10): performed in Mel/L‐Pro eutectogel at 5 wt% at 4°C, for 24 h, on rest.

*n*_a_/*n*_L‐Pro_	Conversion, %^a^	Yield, %^a^	Diastereomeric ratio^b^ (ANTI/SYN; %)	Enantiomeric excess^b^ (ANTI/SYN; %)
1	92	88	78/22	68/15
1.5	90	89	80/20	66/13
1.9	89	86	81/19	67/12
3.8	84	73	74/26	75/25
7.6	77	68	78/22	70/10
11.4	77	64	82/18	61/23
15.2	75	64	82/18	61/23
19	67	59	82/18	73/13
23	68	61	68/32	66/43

a Yield and conversion of the reaction are obtained after purification by chromatographic column; values were reproducible within ± 3%.

b Diastereomeric ratio and enantiomeric excess were determined by using an HPLC with a chiral stationary phase.

In all cases, the gel nature of samples, at the end of the reaction, was verified using the tube inversion test [26]. Gel phases remained stable and supported at least six times the usual amounts of reagents. Analysis of data collected evidences that the highest ratio *n*_a_/*n*_L‐Pro_ that induced a change in conversion and yield values, without affecting stereoselectivity of the reaction, was equal to 15.

To have a better evaluation of the catalytic performance of Mel/L‐Pro eutectogel, results collected in this work were compared with the ones previously reported in the literature for the same reaction performed in catalytic gel systems (Table 9).

**TABLE 9:** Reaction conditions, conversion, yield, and *dr* and *ee* ratios for the aldol reaction between cyclohexanone and *p*‐nitrobenzaldehyde performed in different gel phases.

Gel	*T*, °C	t, h	Yield, %	***dr***_**anti/syn**_ **, %**	*ee*, *%*	Reuse	Reference
Mel/L‐Pro	4	24	73	75/25	75 (anti)	5	This work
L‐Pro	20	24	95	28/72	97 (anti)	5	[16]
Polymorph of L‐Pro hydrogel	25	24	95	14/86	85 (anti)	—	[40]
L‐Pro‐based hydrogel	25	36	99	25/75	12 (anti)	2	[14]
L‐Pro‐based hydrogel	5	24	98	8/92	88 (anti)		[14]
Proline amide	25	4	78	33/67	87 (anti)	4	[41]
Microgel catalyst	25	48	88		82	4	[42]
Gelatin protein	37	168	62	21/79	—	2	[43]
Chitosan hydrogel beads	25	48	85	30/70	—	—	[44]

Aldol reaction has been extensively investigated in literature using hydrogel‐based catalysts, but only few examples deal with the use of eutectogels. In general, yield values ranged from 62% up to 99% (L‐Pro‐based hydrogel) [14], but in this latter case, a larger ketone excess was used (*n*_k_/*n*_a_ = 20). As for the reaction time, Mel/L‐Pro eutectogel proves to be comparable or, in some cases, better than the other catalytic gels previously reported. Comparison with *dr* and *e* *e* values of L‐Pro‐based hydrogel [16], in which reaction was carried out at different temperatures, confirms that lower reaction temperatures provides superior stereoselective control. Most literature reports either do not address catalyst recyclability or show effectiveness for only a limited number of cycles. Only L‐Pro eutectogel maintains its performance in terms of stereoselectivity for five cycles, as Mel/L‐Pro eutectogel studied in this work. This highlights the inherent advantages of utilizing DES as the gelation solvents, as eutectogels are characterized by high structural stability, which significantly facilitates the recovery and reuse of the catalytic phase compared to traditional hydrogel or organogel systems.

## 3 Conclusions

One of the main goals of sustainable chemistry is the development of new systems that combine low environmental impact, high efficiency, and recyclability. In this context, in this work, we explored the possibility to obtain supramolecular Mel/amino acid‐based eutectogels, integrating the convenient properties of DES with the ones of bicomponent gelators that can be obtained from waste. The chiral bicomponent eutectogels were used as heterogeneous catalysts for aldol reaction between *p*‐nitrobenzaldehyde and cyclohexanone.

Soft materials were characterized from a thermal and mechanical point of view. Furthermore, fluorescence and RLS investigations were carried out to have insights about the nature and composition of the gelator, together with information about gelation mechanism and features of aggregates present in gel phases. In general, these analyses pointed out a different behavior depending on the aromatic or aliphatic nature of cogelator. When aliphatic amino acids were used, eutectogels with higher thermal stability, larger aggregates and faster formation were obtained.

Optimization of the aldol reaction identified Mel/L‐Pro eutectogel as the most effective catalyst when reaction was carried out 4°C, for 24 h, on rest. In the above conditions, we observed a predominance of the *anti* stereoisomer, with *dr* and *ee* values reaching 74% and 75%, respectively. Good values of conversion and yield values in eutectogel (84% and 75%) were significantly better than the ones collected in DES solution, under the same experimental conditions, demonstrating the relevance of proximity effects occurring in confined reaction media. The best catalytic system could be reused, for at least five cycles without significant changes in terms of *dr* or *ee* values. Furthermore, it was able to support large amounts of reagents without effect on stereoselectivity and only a modest loss in terms of conversion and yield.

Investigation aimed at analyzing the substrate scope evidenced the target reaction was favored in the presence of aldehydes bearing electron withdrawing groups, whereas the change in the ketone nature shed light on the positive effect exerted by the increase in steric hindrance on the stereoselectivity of the reaction.

Performance of the best catalytic eutectogel was comparable to the ones of most hydrogel systems previously reported in literature, but it represents one of thew few examples so far reported in which eutectogels are used as confined reaction media.

In conclusion, these chiral bicomponent eutectogels prove to be sustainable and efficient catalysts for the aldol reaction, with good performance in terms of conversion and yield, as well as stereoselectivity. Products can be easily obtained performing a liquid–liquid extraction with a green solvent (ethyl acetate), and recycling can be carried out without effect on the stereoselectivity.

## 4 Experimental Section

### 4.1 Materials

Melamine (Merck, 98%), L‐leucine (TCI, 99%), L‐proline (Merck, 99%), L‐phenylalanine (TCI 99%), D‐phenylalanine (Merck, 98%), methanol (Carlo Erba, ACS grade), *p*‐nitrobenzaldehyde (Merck 98%), *p*‐methoxybenzaldehyde (Merck, 98%), cyclohexanone (Merck, 99%), acetone, acetophenone (Merck, 99%), ethyl acetate (Carlo Erba, ACS grade), petroleum ether (Carlo Erba, ACS grade), choline chloride (VWR, >98%), ethylene glycol (Merck, 99%), diethylene glycol (Merck, 99%), and triethylene glycol (Merck, 99%) were obtained from commercial sources and used as received.

### 4.2 Instruments

An Arex 6 Digital Pro plate was used for gelation tests. An IKA C‐MAG HS 7 plate was used for the determination of *T*_gel_. A JASCO FP‐777WI spectrofluorometer was used for the acquisition of emission and excitation spectra and RLS kinetics. SEM images were acquired through a PRO X PHENOM scanning electron microscope. HPLC Shimadzu SIL 40CXR equipped with a temperature controller and UV detector SPD‐40V was used to identify products of aldol reaction. Anton Paar MCR 302E rheometer was used for rheological investigations.

### 4.3 General Procedure for the Preparation of DES

A mixture of choline chloride and hydrogen bond donor was stirred for 30 min at 80°C, obtaining the corresponding DES. After heating, the mixture was held at 60°C under reduced pressure for 1 h using a rotavapor and stored in a desiccator over calcium chloride before usage.

### 4.4 General Procedure for Eutectogel Preparation

The composites were prepared by weighing the suitable amounts of Mel and amino acids and grinding in mortar to obtain a homogeneous powder. Gels were prepared by weighing into a screw‐capped vial (diameter 1 cm) the suitable amounts of composite and 250 mg of DES. The sample vial was heated at 100°C until a clear solution was obtained. Then, the vial was stored at room temperature overnight. Gelation was verified by the tube inversion test [26].

### 4.5 Determination of CGC

Using the same methods described previously, various gelation tests were performed by decreasing the concentration of gelator. The lowest concentration at which gel formation occurs, verified by the tube inversion test, was identified as the CGC.

### 4.6 Determination of T_gel_

*T*_*gel*_ was determined through the falling drop method [27]. The vial, containing the gel, was immersed upside down in a water and ice bath, and the bath temperature gradually increased. The temperature at which the first drop of the liquid fell was recorded as *T*_gel_. Measurements were carried out in triplicate, and these values were reproducible within 1°C.

### 4.7 Emission and Excitation Spectra

Mel/amino acid solutions in DES were prepared by adding different aliquots of Mel and amino acid methanol solutions in a vial; after removing the methanol, under reduced pressure, 600 mg of ChCl/TEG was added. The mixtures were sonicated to obtain solutions in DES. Emission spectra were acquired with a spectrofluorophotometer (JASCO FP‐777W) using a quartz cuvette (light path 0.2 cm). Emission spectra were recorded from 360 to 600 nm, under excitation at 335 nm. The excitation and emission slit widths were set at 3 nm. Normalized emission spectra were obtained by dividing the experimental intensities by the respective intensities at *λ*_max_.

### 4.8 Rheological Measurements

Rheological measurements were carried out on a rheometer on a plate–plate geometry. The diameter of the plates used was 2.5 cm. The gap was kept constant throughout the measurements. Gels were prepared at 5 wt% in 1.5 g of DES. Samples for a typical measurement were prepared by transferring the hot solution into a bowl‐shaped container, in which the gel was allowed to form at room temperature overnight. Then, the gel was transferred on the plate of the rheometer. For each gel, strain and frequency sweeps were carried out three times on three different aliquots. Strain sweeps were recorded at a fixed angular frequency of 1 rad s^−1^ and frequency sweeps at a fixed strain of 0.02%, close to the upper limit of the linear viscoelastic region. To avoid slippage of the sample an upper plate with roughened surface was used. In addition, during the experiments, normal force was monitored, which in all cases remained stable, with neither large negative or positive values, nor sudden variations, indicating consistent sample‐geometry contact. After the experiments, all samples showed no visible evidence of slippage. Thixotropy measurements were carried out by subjecting the sample alternatively to nondestructive strain (*γ* = 0.02%) for 120 s and to destructive strain (*γ *= 20%) for 120 s. This sequence was repeated five times. Angular frequency was maintained at *ω* = 1 rad/s. All measurements were performed at 25°C.

### 4.9 RLS Measurements

RLS measurements were carried out using a spectrofluorophotometer (JASCO FP‐777W) using a synchronous scanning mode in which the emission and excitation monochromators were preset to identical wavelengths. The RLS spectrum was recorded from 400 to 750 nm, with the excitation slit width set at 1.5 nm and emission slit width set at 3 nm. Samples for a typical measurement were prepared by injecting into a quartz cuvette (light path 0.2 cm) the limpid hot solution of gelator. The gel phase obtained at the end of the measurement was stable at the tube inversion test [26].

### 4.10 Scanning Electron Microscopy

SEM images were recorded with an instrument operating at a voltage of 15 kV and under a low vacuum. The gel was placed on the stub and washed with 2 × 50 µL ethyl acetate to remove the DES to obtain the xerogel.

### 4.11 Self‐Sustaining Ability Tests

For a typical test, the gel (1 g) was prepared in a plastic blister, according to the procedure described above. Then, it was transferred by sliding onto a glass coverslip, checking, as a function of time, for any change in shape or collapse.

### 4.12 General Procedure for the Aldol Reaction in Gel Phase

*p*‐Nitrobenzaldehyde (0.132 mmol, 1 eq.) and cyclohexanone (1.32 mmol, 10 eq.) were added on the top of gel (250 mg) in a vial. The reaction mixture was kept at 25°C for 20 h at 120 rpm in an orbital stirrer or at 4°C in the fridge for 24 h on rest. After the suitable time, the reaction mixture was extracted with portion of 300 µL of ethyl acetate until TLC, performed on the organic phase, evidenced the presence of reagents and products. The resulting crude was purified by column chromatography, using a mixture of petroleum ether:ethyl acetate in 7:1 and 5:1 ratio as eluents. In case of gel recycling, after the extraction, the residual ethyl acetate was removed by evaporation under vacuum, and the previously described procedure to perform the reaction was repeated.

### 4.13 General Procedure for Aldol Reaction in DES Solution

L‐Pro (26 mol %) was solubilized in DES, together with 1 eq. of *p*‐nitrobenzaldehyde and 10 eq. of cyclohexanone. The solution was kept in an orbital shaker, at 25°C and 130 rpm or, in the case in which the reaction temperature was equal to 4°C, in a fridge for the suitable time. Then, the reaction mixture was extracted with ethyl acetate until TLC, performed on the organic phase, evidenced the presence of reagents and products. The reaction product was obtained after flash chromatography, as described previously.

### 4.14 Product Identification of Aldol Reaction

Products of aldol reaction were separated by HPLC after dissolution in isopropanol (2 mL). The enantiomeric composition of the product was determined by HPLC analysis (265 nm, Chiralpak AD‐H, flow 0.8 mL/min, eluent n‐hexane/isopropanol = 90:10; *t*_syn_ _major_ = 26 min; *t*_syn_ _minor_ = 29 min; *t*_anti_ _minor_ = 32 min; *t*_anti_ _major_ = 45 min) [45, 46].

## Funding

This study was supported by Università degli Studi di Palermo (FFR2025).

## Conflicts of Interest

The authors declare no conflicts of interest.

## Supporting information

Supplementary Material
