# Supplementary material for: Chiral Bicomponent Melamine/Amino Acid Eutectogels: Efficient Catalysts for the Aldol Reaction
Source: ChemSusChem. 2026 May 22;19(10):e70751. doi: 10.1002/cssc.70751 (PMC13206207; doi:10.1002/cssc.70751)
Supplement: Supplementary file 1 — Supplementary Material [file CSSC-19-e70751-s001.pdf]

Supporting Infgormation for

Chiral bicomponent melamine/amino acid eutectogels: efficient catalysts for the aldol reaction

*Salvatore Marullo, Paolo Reale, Francesca D'Anna**

Università degli Studi di Palermo, Dipartimento STEBICEF, Viale delle Scienze, Ed. 17 90128 Palermo (Italy)

Email: francesca.danna@unipa.it

**Table S1.** Gelation test performed using Mel/amino acid composites in o different DES	Page S2
**Table S2.** Percentage of *G'* recovery after the application of a disruptive strain to Mel/L-Pro (2:1) eutectogel, at 5%wt, in ChCl/TEG.	Page S2
**Figure S1.** Rheological investigation for Mel/ amino acid (2:1) eutectogels at 5% wt in ChCl/TEG. Frequency sweeps were performed at $\gamma\%$ = 0.02. Strain sweeps were performed at ω = 1 rad/s	Pages S3-S4
**Figure S2.** a) Excitation spectra (λ_{em}=400 nm) of Mel (1x10^{-5} M) in ChCl/TEG; b) emission spectra of Mel (1x10^{-5} mol/g) in ChCl/TEG.	Page S4
**Figure S3. a)** Emission spectra of Mel 1×10^{-5} mmol/g at various equivalents of L-Pro; **b)** I_{402} *vs* Equivalents of L-Pro; **c)** Emission spectra of Mel 1×10^{-5} M at various equivalents of L-Phe; **d)** I_{402} *vs* Equivalents of L-Phe; **e)** Emission spectra of Mel 1×10^{-5} M at various equivalents of D-Phe; **f)** I_{402} *vs* Equivalents of D-Phe.	Page S5
**Figure S4.** Emission spectrum of Mel eutectogel, at 5% wt, in ChCl/TEG.	Page S6
**Figure S5.** Emission spectra of eutectogel and, after heating, of hot solutions: a) Mel/L-Leu; b) Mel/L-Phe; c) Mel/D-Phe.	Page S6
**Figure S6.** HLPC Chromatograms	Pages S7-S14

Table S1. Gelation test performed using Mel/amino acid composites in o different DES

Gelator	DES	Range of Mel/amino acid concentration (%wt)	Appearence[a]	CGC (% wt)
**Mel**	ChCl/EG	3-5	I	/
**Mel**	ChCl/DEG	2-5	G	3
**Mel**	ChCl/TEG	2-5	G	4
**Mel/L-Leu 1:1**	ChCl/EG	3-8	S	/
**Mel/L-Leu 1:2**	ChCl/EG	3-8	S	/
**Mel/L-Leu 1:3**	ChCl/EG	3-8	S	/
**Mel/L-Leu 2:1**	ChCl/DEG	3-8	S	/
**Mel/L-Leu 1:1**	ChCl/DEG	5-8	S	/
**Mel/L-Leu 1:2**	ChCl/DEG	5-8	S	/
**Mel/L-Leu 2:1**	ChCl/TEG	2-5	G	3
**Mel/L-Leu 1:1**	ChCl/TEG	2-5	G	4
**Mel/L-Leu 1:2**	ChCl/TEG	2-5	G	5
**Mel/L-Leu 1:3**	ChCl/TEG	2-5	G	8
**Mel/L-Phe 1:1**	ChCl/EG	3-8	G	8
**Mel/L-Phe 1:3**	ChCl/EG	3-5	G	5
**Mel/L-Phe 1:1**	ChCl/DEG	3-8	S	/
**Mel/L-Phe 1:3**	ChCl/DEG	3-5	S	/
**Mel/L-Phe 2:1**	ChCl/TEG	2-5	G	3
**Mel/L-Phe 1:1**	ChCl/TEG	3-5	G	4
**Mel/L-Pro 1:1**	ChCl/EG	3-8	S	/
**Mel/L-Pro 1:3**	ChCl/EG	3-8	S	/
**Mel/L-Pro 1:1**	ChCl/DEG	3-8	S	/
**Mel/L-Pro 1:3**	ChCl/DEG	3-8	S	/
**Mel/L-Pro 2:1**	ChCl/TEG	2-5	G	3
**Mel/L-Pro 1:1**	ChCl/TEG	2-5	G	4
**Mel/D-Phe 2:1**	ChCl/TEG	2-5	G	3

a) Appearance: S=soluble I= insoluble G=gel

Table S2. Percentage of *G'* recovery after the application of a disruptive strain to Mel/L-Pro (2:1) eutectogel, at 5%wt, in ChCl/TEG.

Cycle	% recovery G'
I	38
II	65
III	77
IV	87

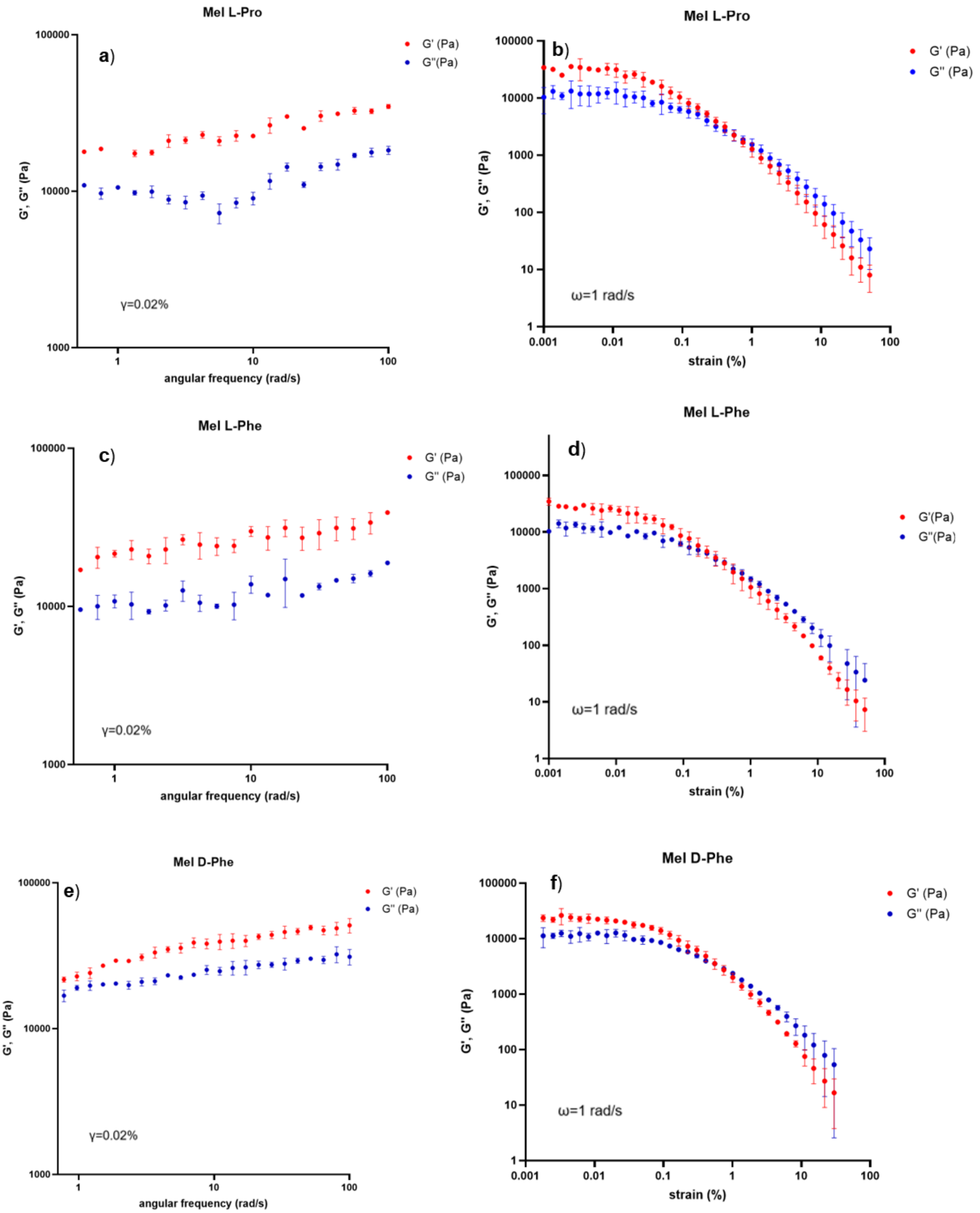

Figure S1. Rheological investigation for Mel/ amino acid (2:1) eutectogels at 5% wt in ChCl/TEG**.** Frequency sweeps were performed at γ% = 0.02. Strain sweeps were performed at ω = 1 rad/s

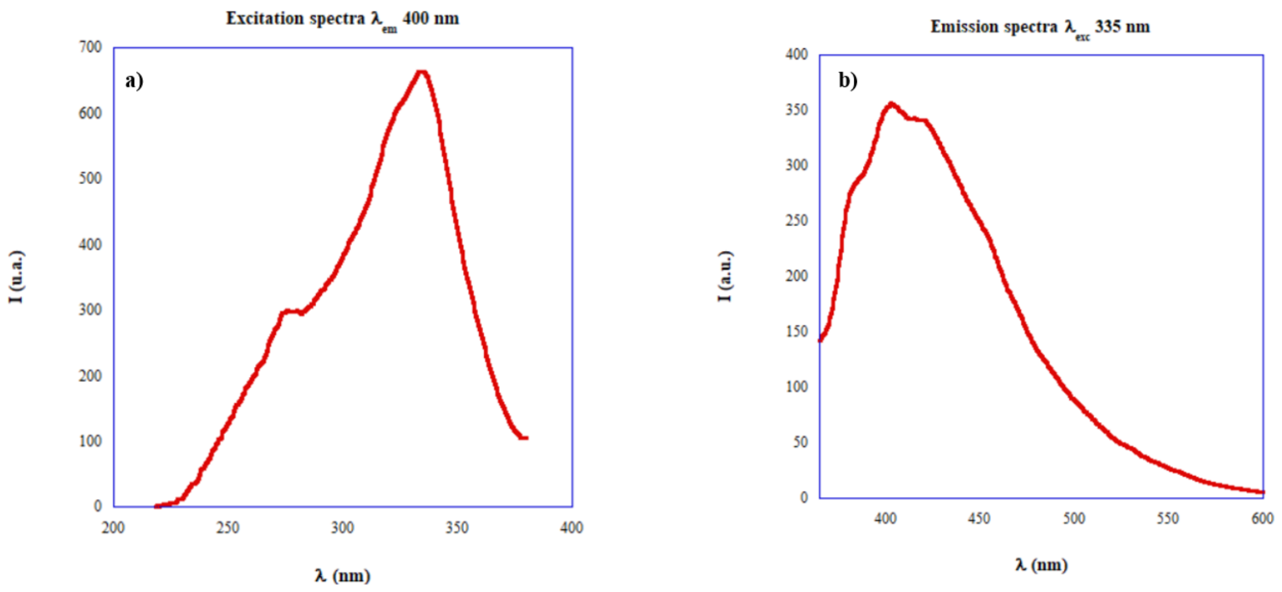

Figure S2. **a)** Excitation spectra (λ_{em}=400 nm) of Mel (1x10^{-5} M) in ChCl/TEG; **b)** emission spectra of Mel (1x10^{-5} mol/g) in ChCl/TEG.

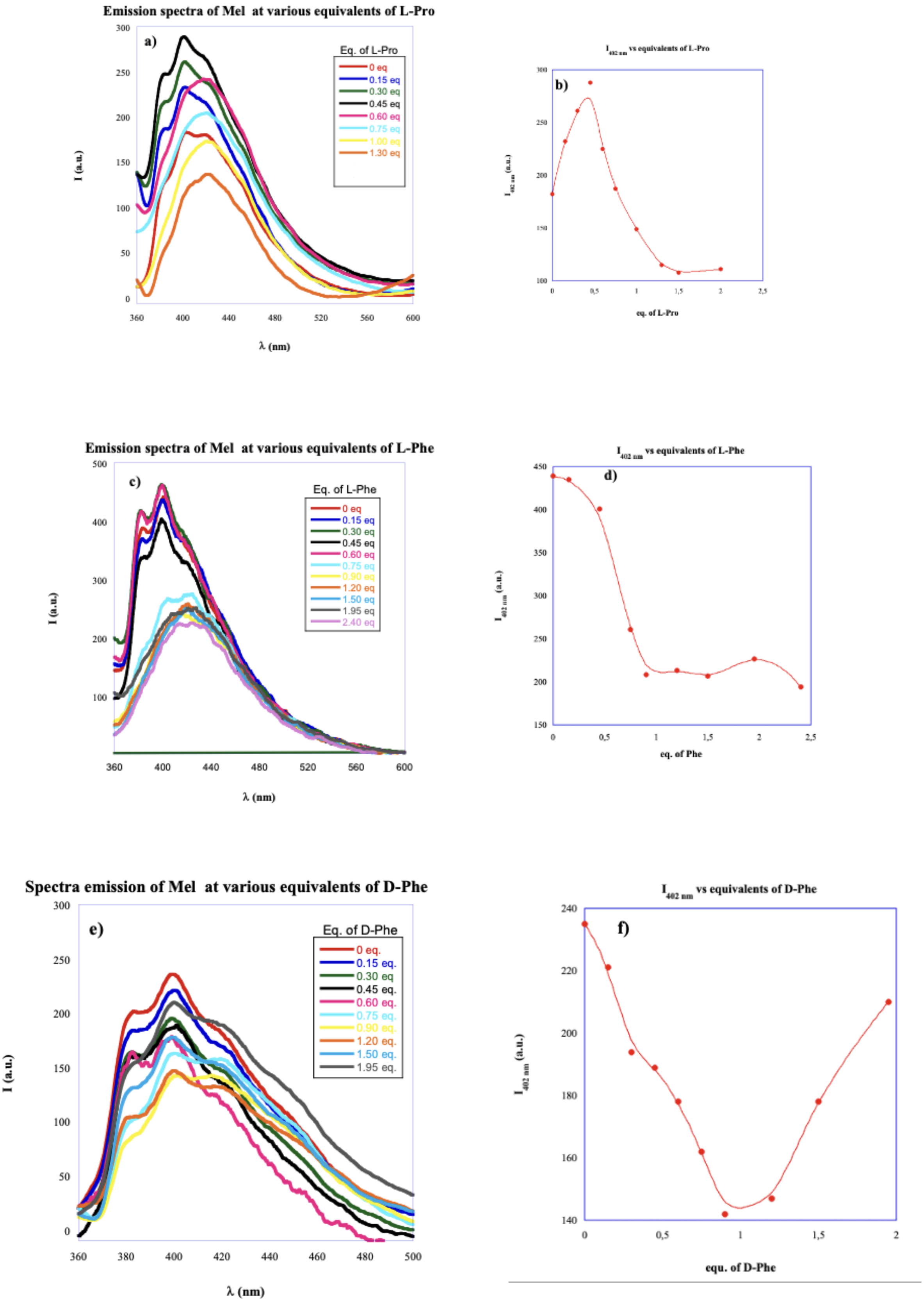

Figure S3. a) Emission spectra of Mel 1×10^{-5} mmol/g at various equivalents of L-Pro; **b)** I_{402} *vs* Equivalents of L-Pro; **c)** Emission spectra of Mel 1×10^{-5} M at various equivalents of L-Phe; **d)** I_{402} *vs* Equivalents of L-Phe; **e)** Emission spectra of Mel 1×10^{-5} M at various equivalents of D-Phe; **f)** I_{402} *vs* Equivalents of D-Phe.

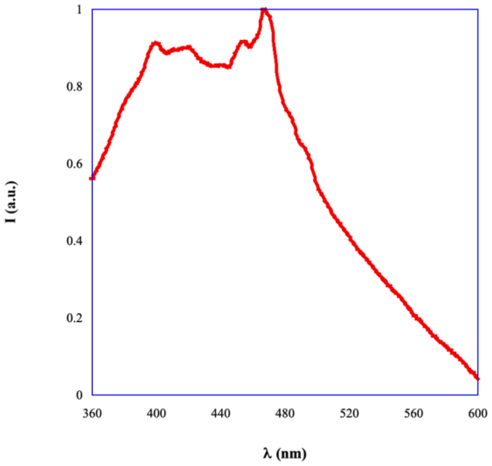

Figure S4. Emission spectrum of Mel eutectogel, at 5% wt, in ChCl/TEG.

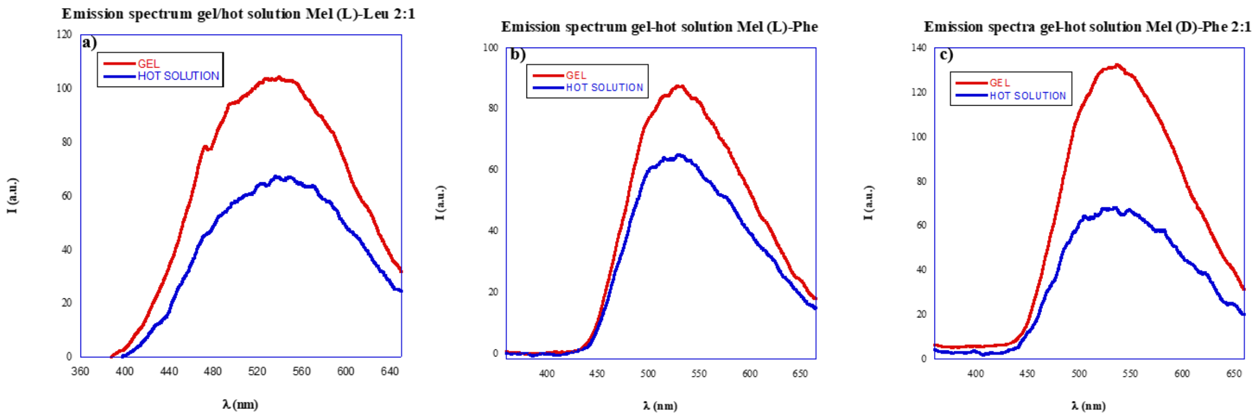

Figure S5. Emission spectra of eutectogel and, after heating, of hot solutions: **a)** Mel/L-Leu; **b)** Mel/L-Phe; **c)** Mel/D-Phe.

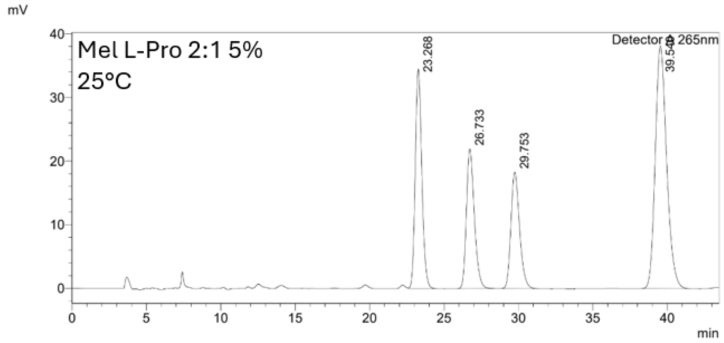

Peak#	Ret. Time	Area	Height	Conc.
1	23.268	1055984	34525	22.759
2	26.733	814941	21952	17.564
3	29.753	739255	18303	15.933
4	39.540	2029590	38133	43.743
Total		4639771	112912	

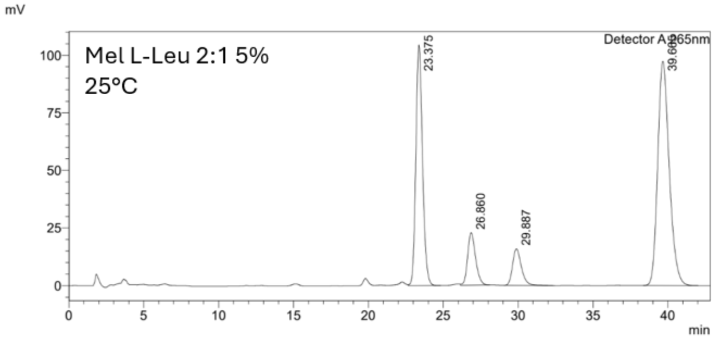

Peak#	Ret. Time	Area	Height	Conc.
1	23.375	3213651	104396	32.395
2	26.860	842889	22779	8.497
3	29.887	654892	15888	6.602
4	39.665	5208760	97318	52.507
Total		9920191	240381	

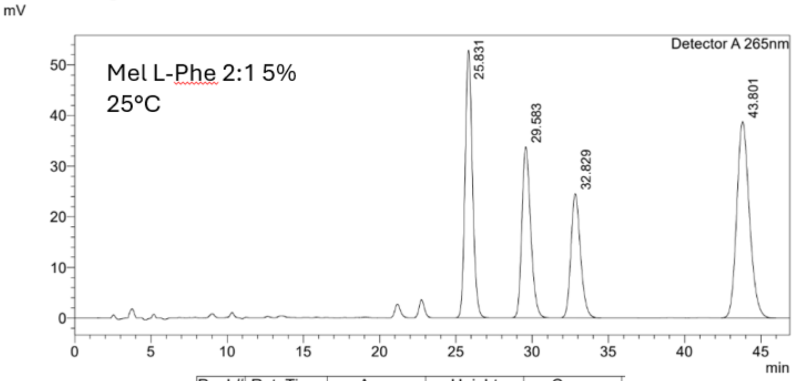

Peak#	Ret. Time	Area	Height	Conc.
1	25.831	1767607	52812	27.495
2	29.583	1351412	33743	21.021
3	32.829	1069678	24524	16.639
4	43.801	2240214	38780	34.846
Total		6428911	149860	

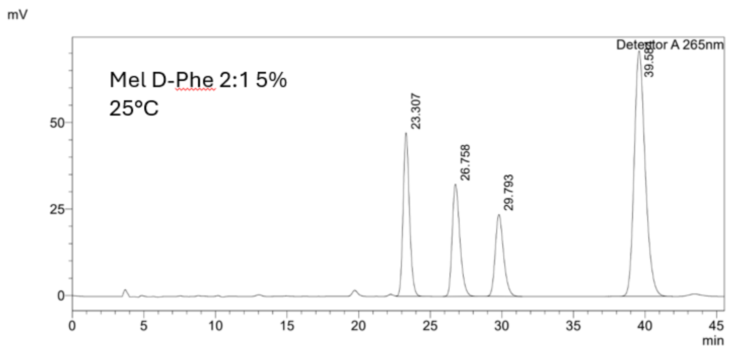

Peak#	Ret. Time	Area	Height	Conc.
1	26.297	12515655	244930	33.099
2	29.461	3840362	89659	10.156
3	30.670	3440226	77240	9.098
4	39.563	18016366	267256	47.646
Total		37812610	679084	

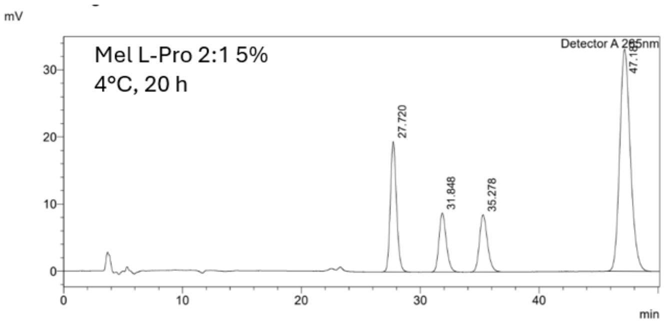

Peak#	Ret. Time	Area	Height	Conc.
1	27.720	697775	19442	19.802
2	31.848	372007	8798	10.557
3	35.278	395511	8513	11.224
4	47.181	2058426	33135	58.416
Total		3523719	69888	

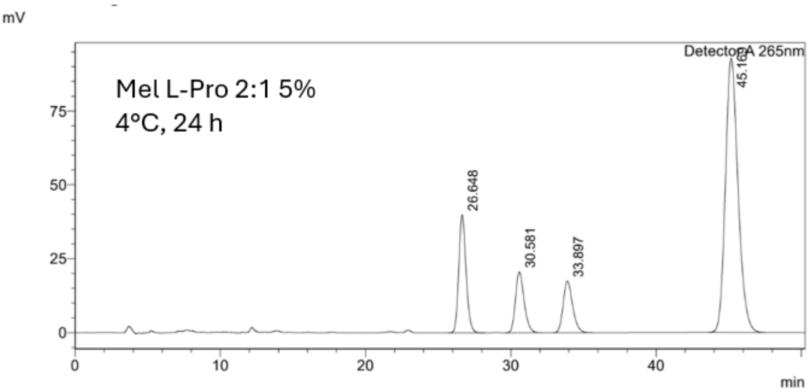

Peak#	Ret. Time	Area	Height	Conc.
1	26.648	1399129	40038	16.190
2	30.581	854437	20584	9.887
3	33.897	792047	17456	9.165
4	45.169	5596160	92837	64.757
Total		8641772	170915	

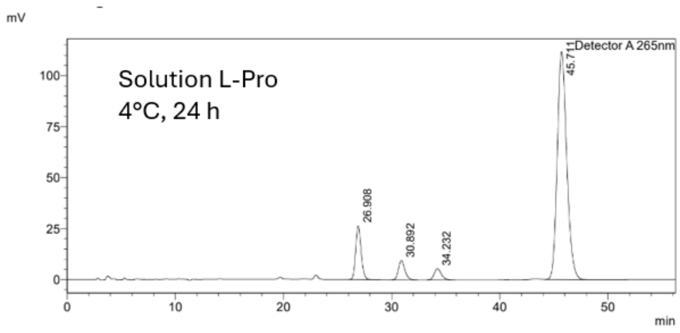

Peak#	Ret. Time	Area	Height	Conc.
1	26.908	924908	26359	11.042
2	30.892	395111	9494	4.717
3	34.232	255799	5564	3.054
4	45.711	6800154	111868	81.186
Total		8375972	153285	

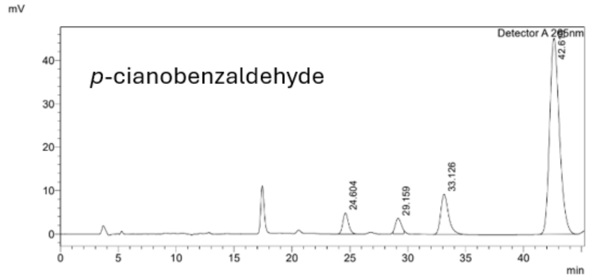

Peak#	Ret. Time	Area	Height	Conc.
1	24.604	162612	4830	4.820
2	29.159	126660	3551	3.754
3	33.126	437902	9280	12.979
4	42.619	2646778	45235	78.447
Total		3373953	62896	

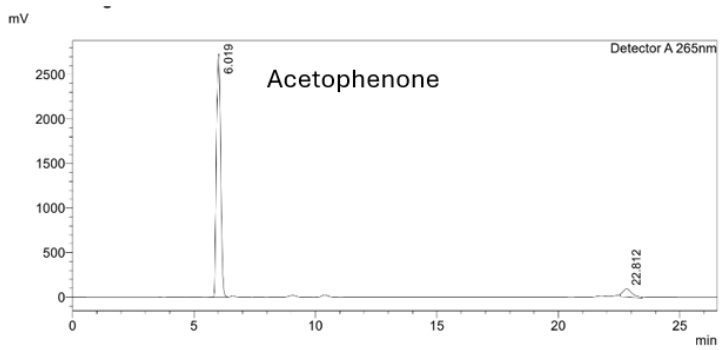

Peak#	Ret. Time	Area	Height	Conc.
1	6.019	33632311	2727060	93.060
2	22.812	2508238	91674	6.940
Total		36140549	2818734	

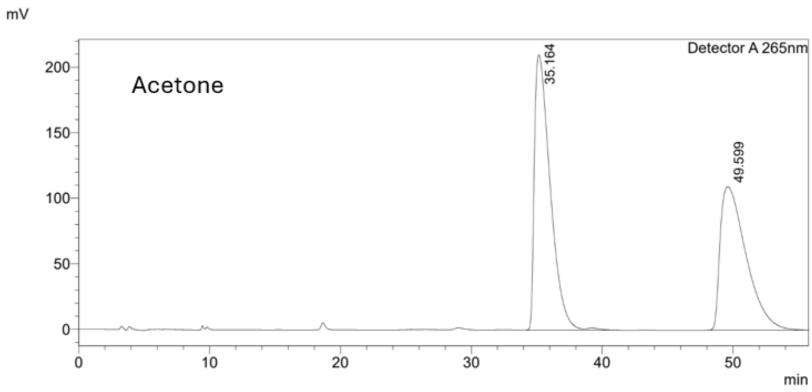

Peak#	Ret. Time	Area	Height	Conc.
1	35.164	17719059	210121	54.122
2	49.599	15019974	109443	45.878
Total		32739033	319564	

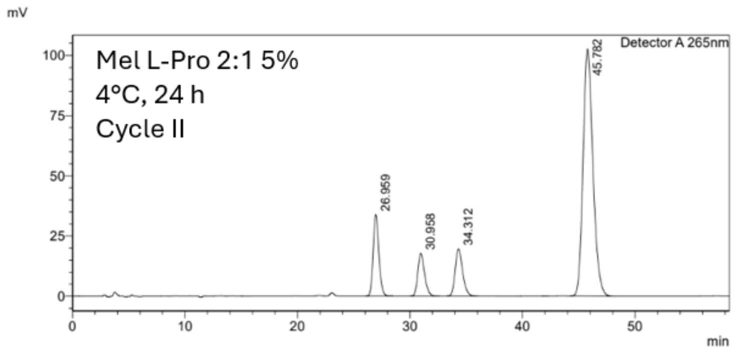

Peak#	Ret. Time	Area	Height	Conc.
1	26.959	1192301	33779	13.076
2	30.958	747288	17754	8.196
3	34.312	903392	19614	9.908
4	45.782	6275130	102629	68.820
Total		9118112	173776	

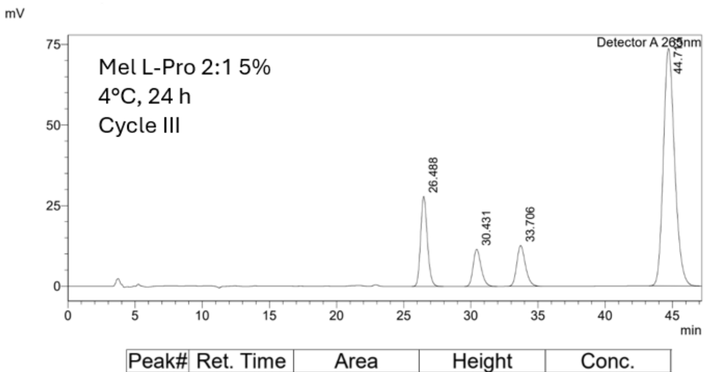

Peak#	Ret. Time	Area	Height	Conc.
1	26.488	969080	27934	15.342
2	30.431	474904	11522	7.518
3	33.706	566433	12616	8.967
4	44.713	4306277	73674	68.173
Total		6316694	125745	

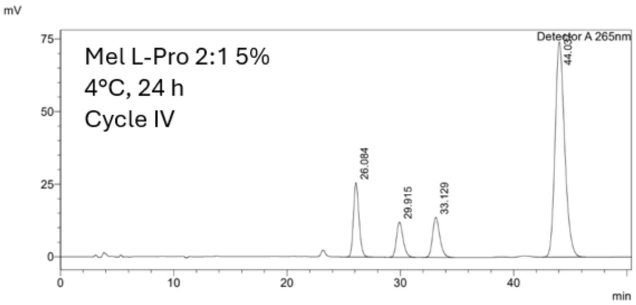

Peak#	Ret. Time	Area	Height	Conc.
1	26.084	870902	25606	13.786
2	29.915	491965	12129	7.788
3	33.129	608602	13744	9.634
4	44.037	4345893	74034	68.793
Total		6317362	125513	

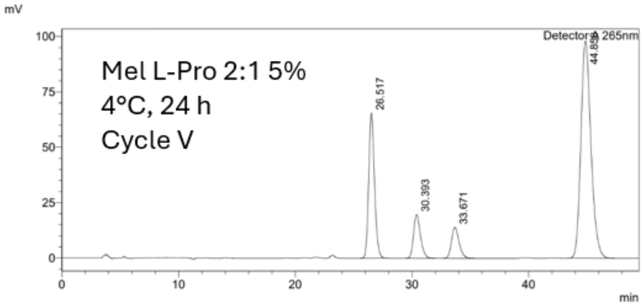

Peak#	Ret. Time	Area	Height	Conc.
1	26.517	2272835	65558	23.684
2	30.393	813992	19710	8.482
3	33.671	632209	14017	6.588
4	44.858	5877339	98072	61.245
Total		9596375	197357	

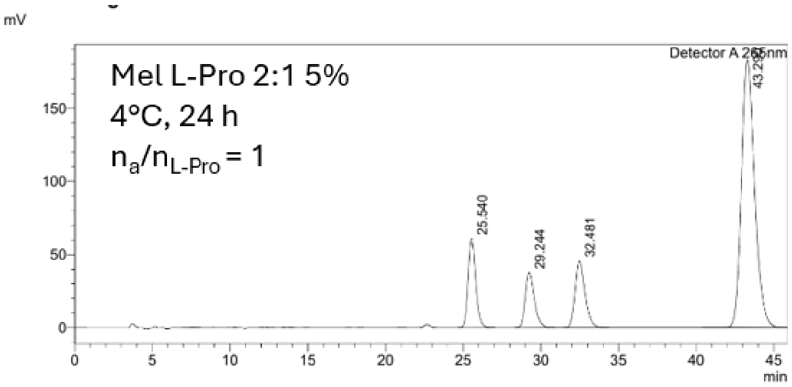

Peak#	Ret. Time	Area	Height	Conc.
1	25.540	2053923	60663	12.544
2	29.244	1523205	37485	9.302
3	32.481	2022009	45509	12.349
4	43.290	10775060	183097	65.805
Total		16374196	326754	

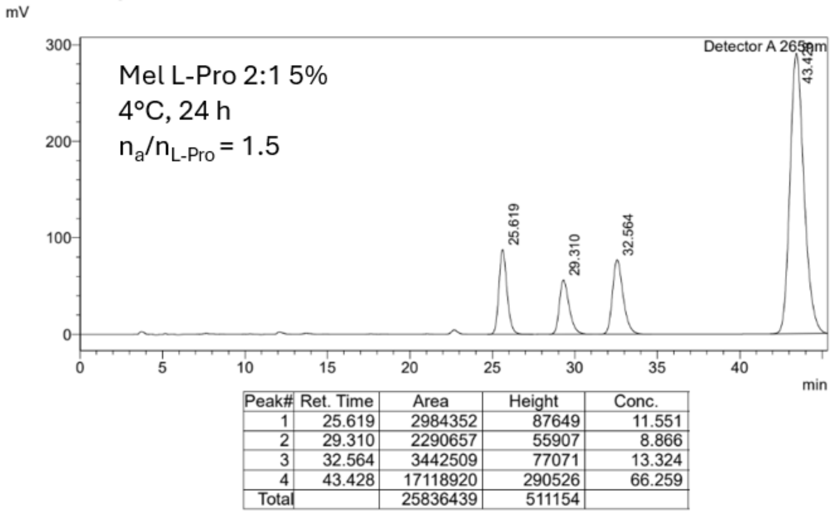

Peak#	Ret. Time	Area	Height	Conc.
1	25.619	2984352	87649	11.551
2	29.310	2290657	55907	8.866
3	32.564	3442509	77071	13.324
4	43.428	17118920	290526	66.259
Total		25836439	511154	

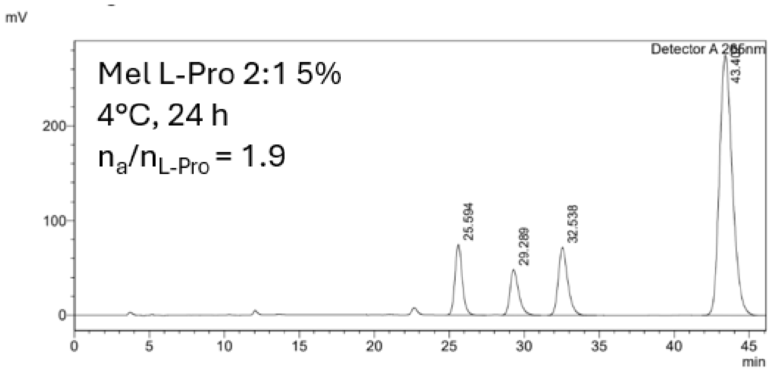

Peak#	Ret. Time	Area	Height	Conc.
1	25.594	2538801	74590	10.584
2	29.289	1981700	48247	8.261
3	32.538	3205483	71751	13.363
4	43.402	16262062	274951	67.792
Total		23988046	469538	

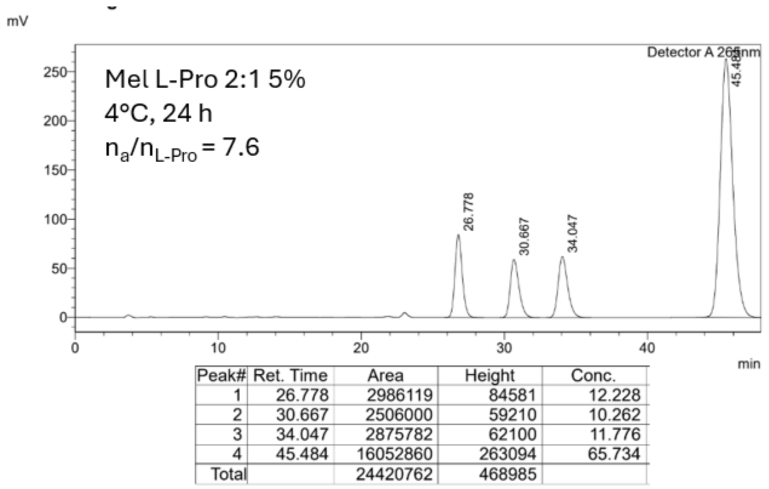

Peak#	Ret. Time	Area	Height	Conc.
1	26.778	2986119	84581	12.228
2	30.667	2506000	59210	10.262
3	34.047	2875782	62100	11.776
4	45.484	16052860	263094	65.734
Total		24420762	468985	

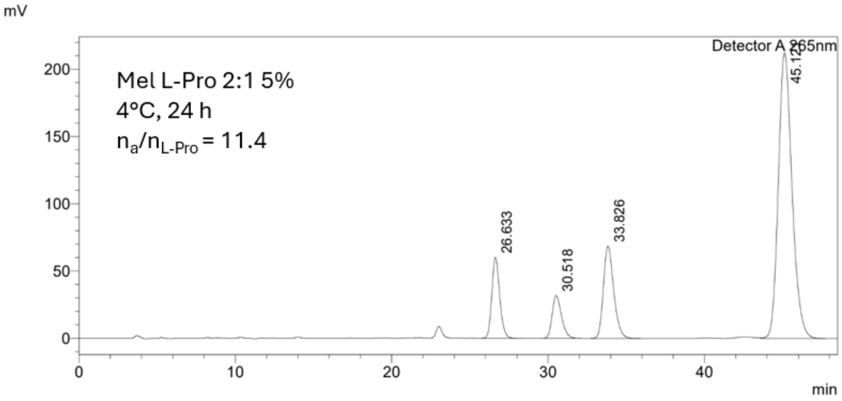

Peak#	Ret. Time	Area	Height	Conc.
1	26.633	2108569	60204	10.823
2	30.518	1324357	31779	6.798
3	33.826	3151304	68714	16.175
4	45.121	12898264	212400	66.204
Total		19482494	373098	

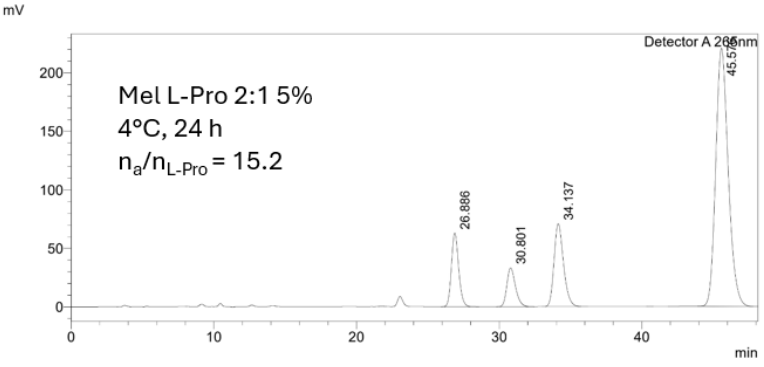

Peak#	Ret. Time	Area	Height	Conc.
1	26.886	2223976	62999	10.927
2	30.801	1395145	33190	6.855
3	34.137	3270005	70857	16.067
4	45.576	13463779	220561	66.152
Total		20352905	387607	

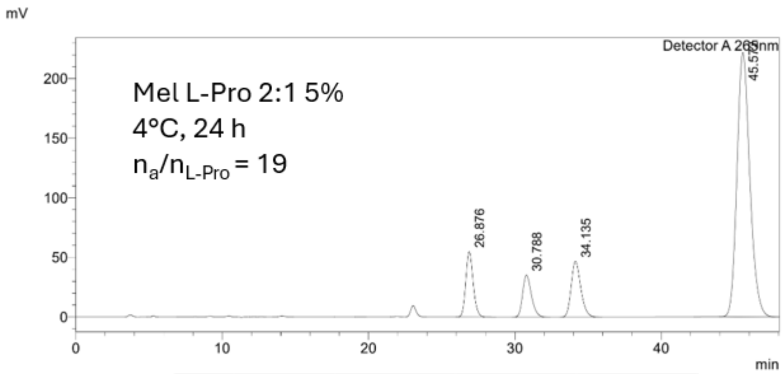

Peak#	Ret. Time	Area	Height	Conc.
1	26.876	1935506	54920	10.127
2	30.788	1481209	35314	7.750
3	34.135	2153904	46861	11.270
4	45.572	13541907	221915	70.854
Total		19112526	359010	

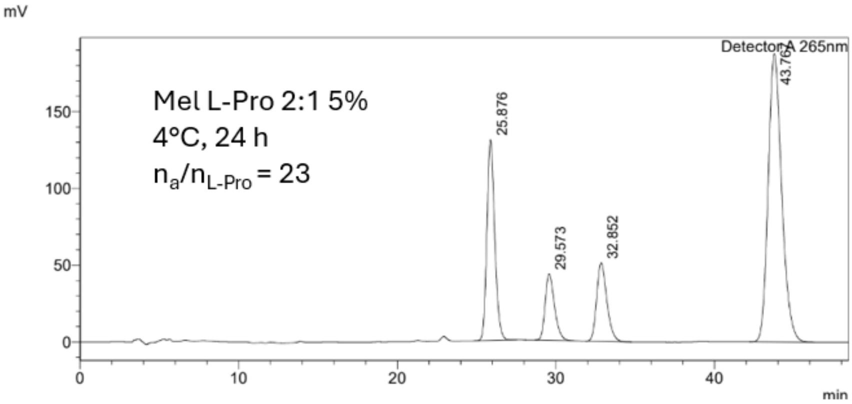

Peak#	Ret. Time	Area	Height	Conc.
1	25.876	4482748	130804	22.788
2	29.573	1774334	43173	9.020
3	32.852	2288654	51163	11.635
4	43.767	11125368	187554	56.557
Total		19671104	412695	

Figure S6. HLPC Chromatograms
